# Phonon engineering in Yb:La_2_CaB_10_O_19_ crystal for extended lasing beyond the fluorescence spectrum

**DOI:** 10.1038/s41377-023-01243-x

**Published:** 2023-08-25

**Authors:** Yanling Cheng, Fei Liang, Dazhi Lu, Jingcheng Feng, Guochun Zhang, Haohai Yu, Huaijin Zhang, Yicheng Wu

**Affiliations:** 1 State Key Laboratory of Crystal Materials and Institute of Crystal Materials, Shandong University, Jinan, 250100 China; 2 Key Lab Functional Crystals and Laser Technology, Technical Institute of Physics and Chemistry, Chinese Academy of Sciences, Beijing, 100190 China

**Keywords:** Solid-state lasers, Optical physics

## Abstract

Since the first invention of the laser in 1960, direct lasing outside the fluorescence spectrum is deemed impossible owing to the “zero-gain” cross-section. However, when electron-phonon coupling meets laser oscillation, an energy modulation by the quantized phonon can tailor the electronic transitions, thus directly creating some unprecedented lasers with extended wavelengths by phonon engineering. Here, we demonstrate a broadband lasing (1000–1280 nm) in a Yb-doped La_2_CaB_10_O_19_ (Yb:LCB) crystal, far beyond its spontaneous fluorescence spectrum. Numerical calculations and in situ Raman verify that such a substantial laser emission is devoted to the multiphonon coupling to lattice vibrations of a dangling “quasi-free-oxygen” site, with the increasing phonon numbers step-by-step (*n* = 1–6). This new structural motif provides more alternative candidates with strong-coupling laser materials. Moreover, the quantitative relations between phonon density distribution and laser wavelength extension are discussed. These results give rise to the search for on-demand lasers in the darkness and pave a reliable guideline to study those intriguing electron-phonon-photon coupled systems for integrated photonic applications.

## Introduction

Electron and phonon are two fundamental particles (quasi-particles) of condensed matter and their interplay in single crystals can create many interesting physical phenomena, such as BCS superconductor^1,2^, Jahn–Teller distortion^3^, polariton^4,5^, upconversion fluorescence^6–8^, etc. In a laser crystal, the electronic transitions of active ion can be manipulated by its surrounding lattice vibrations, thereby, the emitting photon energy gradually decreases or increases by the creation or annihilation of quantized phonon^9–11^. At this time, the phonon acts as a crucial unit for tailoring electronic transitions and corresponding fluorescence emission with homogeneous spectral broadening. Therefore, some broadband laser wavelengths can be designed by the selective amplification of phonon-assisted emission^12–14^, which is more convenient than traditional nonlinear optical technology suffering from complex configuration and high cost. In the past 40 years, some broadband vibronic lasers with great spectral broadening were discovered, including Ti:Sapphire^15^, alexandrite^16^, Cr:LiSAF^17^, Tm:CALYO^18^, Yb:YAG^19^, and so on. However, all these laser wavelengths still locate inside the spontaneous fluorescence spectrum, or slightly outside with a few nanometers^20,21^. Therefore, it is still a great challenge to realize lasers far beyond the fluorescence spectrum of the gain materials^22,23^.

In multiphonon-assisted emission, the creation of high-order phonons can reduce photon energy step-by-step, thus pushing laser wavelengths far beyond the spontaneous fluorescence spectrum. This represents a new diagram to search for light in the darkness^24^. Recently, our group demonstrated such a multiphonon-assisted lasing in Yb:YCa_4_O(BO_3_)_3_ (Yb:YCOB) crystal with phonon number *n* = 3–8^9^. A crucial motif “free-oxygen” to strengthen the electron-phonon coupling effect was verified in experiments^25^. However, borates containing “free-oxygen” are very rare, less than one percent of the total rare-earth borates, thereby giving a limitation for searching more strong-coupling laser materials. More importantly, owing to the large energy-level splitting of Yb^3+^ ion in Yb:YCOB, the low-level phonon-assisted transitions are overlapped by direct transitions between electronic energy levels. For example, the maximum Stark splitting of Yb:YCOB is 1022 cm^−1^, totally covering the one-phonon and two-phonon coupling of “free-oxygen” site (average phonon energy ~476 cm^−1^) and partially overlapped with three-phonon coupling process. As a result, it is difficult to distinguish the weak low-order phonon-assisted transition from a broadband spontaneous emission, thus leading to the limitation of multiphonon-assisted lasing with extended wavelengths. Therefore, searching for new structural motifs in rare-earth borates is very essential to elaborate the physics mechanism of multiphonon coupling lasing, and also find their distinctive applications in our life.

Yb-doped La_2_CaB_10_O_19_ (Yb:LCB) crystal is a multi-functional laser crystal^26^. Compared to Yb:YCOB, its Stark splitting is only 640 cm^−1^, being beneficial to avoid overlapping from electronic transitions and separate pure phonon-assisted ones^27^. Herein, we present a broadband laser emission in Yb:LCB crystal beyond its fluorescence spectrum, corresponding to the increasing phonon number *n* = 1–6. The electron-phonon coupling intensity is calculated by the Huang–Rhys *S* factor and the multiphonon-assisted fluorescence lineshape is predicted numerically. Theoretical calculations show that such a substantial lasing spectrum is devoted to multiphonon coupling at a dangling “quasi-free-oxygen” site, as demonstrated by the in situ Raman experiment. Our results represent a significant step forward for phonon-assisted vibronic lasers and provide a possible coherent source for many applications, e.g., Ho^3+^ pump source^28^, upconversion fiber laser^29^, and dermatology^30^.

## Results

**Fig. 1:**
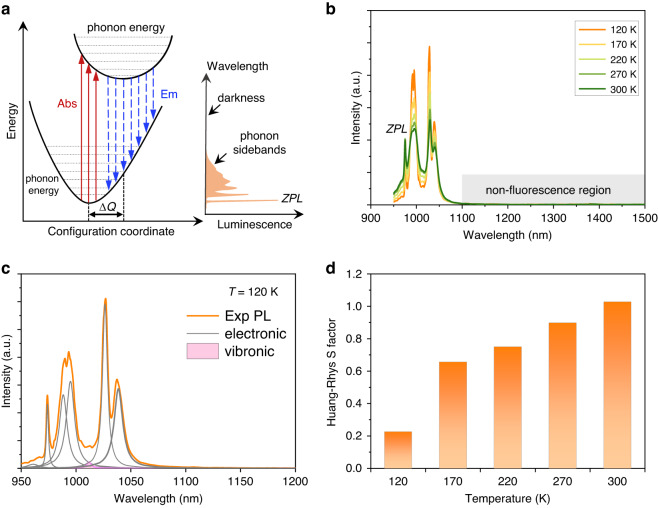
Temperature-dependent fluorescence of Yb:LCB crystal. **a** Configuration model of electron-phonon coupling vibronic process. The ordinate refers to the energy and the abscissa refers to the distance between nucleus. Two parabolas refer to the potential energy curves of the ground state and excited state, respectively, where the excited state parabola is drawn in such a way that the force constant is weaker than in the ground state. Under the Franck–Condon approximation, the vertical red arrows represent the light absorption and the blue arrows represent the fluorescence emission. ZPL is the zero-phonon line. Phonon sidebands represent low-order phonon-assisted emission. Darkness represents no fluorescence signal with multiphonon-assisted transitions. **b** The temperature-dependent fluorescence spectra of Yb:LCB crystal from 120 to 300 K. **c** The peak fitting results of fluorescence spectrum at 120 K. The pink region represents the phonon-assisted emission. **d** Calculated Huang–Rhys factors at various temperatures

First, let us start with a classical configurational model of phonon-assisted vibronic transitions. As depicted in Fig. 1a, in a typical vibronic process, the participation of phonons in the electronic transitions will produce lattice relaxation^31,32^, resulting in the central shift (ΔQ) of ground states and excited states in the electronic energy level. Optical absorption corresponds to a transition from the ground state to the excited state and fluorescence emission is the reverse transition. According to the Schrodinger equation, lattice relaxation means the orthogonality destruction of lattice vibration wave function, from which the participated phonon number in the optical process is not constrained by the lattice symmetry^33^. Therefore, both optical absorption and emission process are associated with the creation and annihilation of many quantized phonons. Such a giant energy transfer can greatly tailor the photon energy of fluorescence emission. In 1950, Huang and Rhys proposed a dimensionless *S* factor to characterize the strength of the electron-phonon coupling in solid materials^31^. It can be calculated by the ratio *W*_*0*_ of the zero-phonon line (ZPL) integral intensity *I*_ZPL_ to the total integral intensity *I* of the fluorescence spectrum, with W_0_ = exp (-S). The fluorescence lineshape function can be predicted by Huang–Rhys theory, where the phonon-assisted fluorescence reaches the maximum when phonon number n = S and gradually decreases when n > S. Therefore, in rare-earth crystals (S < 2), ZPL dominates and the phonon-assisted transitions are very weak. In general, only those few-phonon processes can be observed in the spontaneous emission.

Figure 1b displays the fluorescence spectrum of Yb:LCB crystal under various temperatures from 120 to 300 K. According to the energy-level splitting of Yb^3+^ ion, there are five emission peaks of pure electronic transitions at 976, 990, 994, 1030, and 1041 nm, corresponding to the transition from ^2^F_5/2_ to ^2^F_7/2_ level (Supplementary Fig. 1). The maximum Stark splitting of ^2^F_7/2_ level is 640 cm^-1^. Its Huang–Rhys factor is calculated by spectral fitting, from which the total fluorescence can be divided into electronic peaks and vibronic peaks, where the latter devotes the contribution of phonon-assisted transitions. Regarding the 976 nm as ZPL, the Huang–Rhys *S* factor of Yb:LCB crystal is 0.23 at 120 K (Fig. 1c). With increasing temperatures, the phonon-assisted emission intensities become strong and the corresponding *S* factors gradually increase to 1.02 at 300 K (Fig. 1d and Supplementary Fig. 2). This value is slightly smaller than that of Yb:YCOB crystal (S = 1.34 at 300 K)^9^, indicating a reduced electron-phonon coupling intensity of Yb:LCB. The predicted fluorescence lineshape exhibits that the vibronic emission reaches a maximum when *n* = 1 and dramatically decrease with *n* ≥ 2. Therefore, these high-order phonon-assisted transitions are not observable and there is a broad ‘darkness’ regime at the long wavelength beyond 1100 nm for Yb:LCB crystal.

Now, an emerging question is what happens in the darkness with a large phonon number n » S. In theory, multiphonon-assisted transitions, no matter how extremely weak, always exist. So, it is possible to amplify those weak emissions in the resonant cavity and make it actually lasing. However, the main challenge for such lasing is that multiphonon-assisted transitions are much weaker compared to the overwhelming pure electronic transitions. Therefore, the traditional lasing between direct electronic transitions at 1030 nm and 1041 nm must be suppressed. Then, lasing outside the fluorescence spectrum could be obtained by the amplification of high-order phonon-involved transitions step-by-step.

**Fig. 2:**
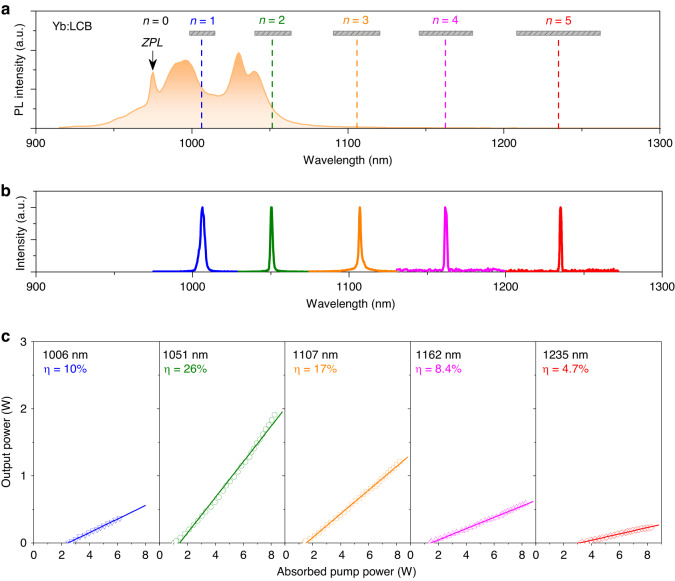
Multiphonon-assisted lasing performances of Yb:LCB crystal. **a** Fluorescence spectrum of Y-cut Yb:LCB at room temperature. The wavelength of the excitation source is 880 nm. **b** Laser spectrum with different phonon numbers *n* = 1–5. λ = 1006 nm (threshold *P*_th_ = 2.50 W), λ = 1051 nm (*P*_th_ = 1.06 W), λ = 1107 nm (*P*_th_ = 1.43 W), λ = 1162 nm (*P*_th_ = 1.51 W), λ = 1235 nm (*P*_th_ = 3.27 W). **c** Laser output power versus the absorbed pump power for various phonon numbers. The solid lines are linear fitting lines. Our used sample is 10 at.% Y-cut Yb:LCB crystal

This “suppression-amplification” strategy could be satisfied in our designed cavity simultaneously, where the target laser wavelength can oscillate in the cavity, but the conventional fluorescence emission is high transmittance on the cavity mirror to suppress laser oscillation. The laser setup is plotted in Supplementary Fig. 4. By designing resonant cavities with a judicious coating (Methods), we realize the multiphonon-assisted lasing beyond the fluorescence spectrum of Yb:LCB crystal. Figure 2 shows several laser wavelengths at 1006, 1051, 1107, 1162, and 1235 nm, corresponding to phonon numbers of *n* = 1–5, respectively. It is observed that all these laser wavelengths don’t locate at strong Stark-transition lines, indicating that they should be devoted to phonon-assisted lasing processes, not the spectral broadening effect induced by heat. Moreover, we also try to obtain lasing at longer wavelengths with *n* ≥ 6. Some laser spectra are observed around 1270–1280 nm (Supplementary Fig. 5), but no effective output power is measured due to the extremely weak multiphonon coupling effect at these wavelengths.

The output powers of various lasers are plotted in Fig. 2c. At 1006 nm, the maximum output power is 372 mW under an absorbed pump power of 6.17 W, corresponding to a slope efficiency of 10%. For *n* = 1, its efficiency should be maximum in Huang–Rhys theory, but here a low efficiency can be attributed to the reabsorption effect at 1006 nm (Supplementary Fig. 6). For *n* = 2, a maximum output power of 1.91 W at 1051 nm is obtained under an absorbed pump power of 8.26 W, corresponding to a slope efficiency of 26%, which is lower than previous 1030 nm laser (η = 50.1%) due to the ‘low-gain’ phonon sideband emission^34^. For *n* = 3, a maximum output power reaches to 1.21 W at 1107 nm, corresponding to a slope efficiency of 17%. For *n* = 4, a maximum output power reaches to 582 mW at 1162 nm, corresponding to a slope efficiency of 8.4%. For *n* = 5, a maximum output power reaches to 232 mW at 1235 nm, corresponding to a slope efficiency of 4.7% and an increased lasing threshold of 3.27 W.

In addition, we measured the fundamental characteristics of multiphonon-assisted Yb:LCB laser beyond the fluorescence spectrum comprising beam quality, stability, polarization, linewidths, etc. (Supplementary Figs. 7–18 and Methods). The temperature-dependent output powers of Yb:LCB crystal show that the laser performance slightly deteriorates with increasing temperature. For the 1107 nm laser, with the temperature increasing from 10 to 50 °C, the threshold of pump power increases from 1.71 to 2.12 W, while the slope efficiency reduces from 17.5 to 16.5%. For the 1162 nm laser, the temperature-dependent threshold of pump power increases from 1.46 to 2.30 W, and the slope efficiency reduces from 8.4 to 7.4%. The deteriorated laser performance could be attributed to the large population at a low level under high temperatures, thereby leading to the difficulty for population inversion associated with the increased threshold. These results indicate that the multiphonon-assisted Yb^3+^-lasers exhibit a strong temperature dependence at the wavelength outside the fluorescence spectrum.

**Fig. 3:**
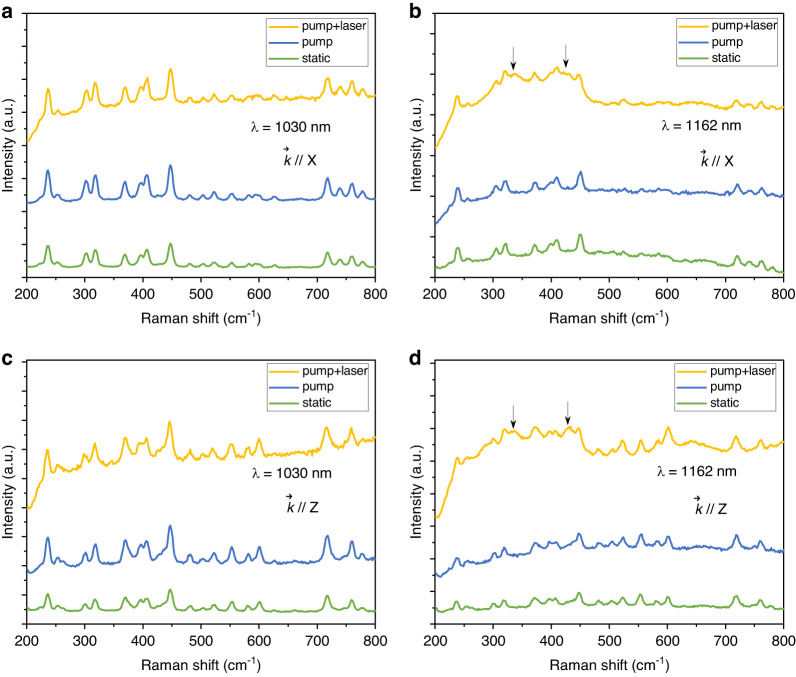
**a**, **c** The $\vec{k}//X$ and $\vec{k}//Z$ in situ Raman measurements of pure electronic transitions at 1030 nm, respectively. **b**, **d** The $\vec{k}//X$ and $\vec{k}//Z$ in situ Raman measurements of lasing beyond the fluorescence at 1162 nm, respectively. “Static” means a normal Raman measurement excited by a He-Ne laser. “Pump” means only the LD-pump light is incident into crystal along the Y-direction without laser operation. “pump + laser” means the LD-pump light is incident into crystal along the Y-direction with laser operation at 1030 nm or 1162 nm. $\vec{k}$ represent the incident direction of the He-Ne laser

The next question is which phonon mode participated in the multiphonon coupling process. In order to elaborate the active phonon modes and numbers, we perform the in situ Raman measurements, as depicted in Fig. 3. The experimental setup is plotted in Supplementary Fig. 19. We set three different measurable conditions, “static”, “pump”, and “pump + laser”, respectively. When some new vibrational peaks appear on Raman spectroscopy with laser running, they will be assigned to the active phonon modes involved in the multiphonon coupling. First, we perform the testing of the laser at 1030 nm. One can see that there are no significant changes in the Raman spectroscopy in all three conditions, suggesting that lasing at 1030 nm is a pure electronic transition process. In comparison, when the laser at 1162 nm is running, there are two enhanced vibrational bands around about 335 and 424 cm^−1^, corresponding to the active phonon modes in phonon-assisted transitions. If rotating the crystal along the Y-axis by 90°, these two enhanced bands still exist, and the peak positions maintain unchanged. Moreover, when the laser is stopped, these two peaks become inactive under the “static” and “pump” conditions. So, we can assign these two vibrational bands to active phonon modes in the multiphonon-coupled lasing, but not static and pump-induced lattice vibrations.

Based on the two vibrational frequencies, we can estimate the involved phonon numbers in the electron-phonon coupling process. Taking the ZPL at 976 nm as *n* = 0, we can deduce that the frequency shift of the 1006 nm laser is 306 cm^−1^, devoting to one-phonon coupling process (*n* = 1). Similarly, the frequency shift of the 1051 nm laser is 731 cm^−1^, corresponding to two-phonon coupling (*n* = 2), where the 1107, 1162, and 1235 nm lasers could be assigned to *n* = 3, *n* = 4, and *n* = 5 cases, respectively. A comprehensive analysis is listed in Supplementary Table S1. This is consistent with our experimental results.

**Fig. 4:**
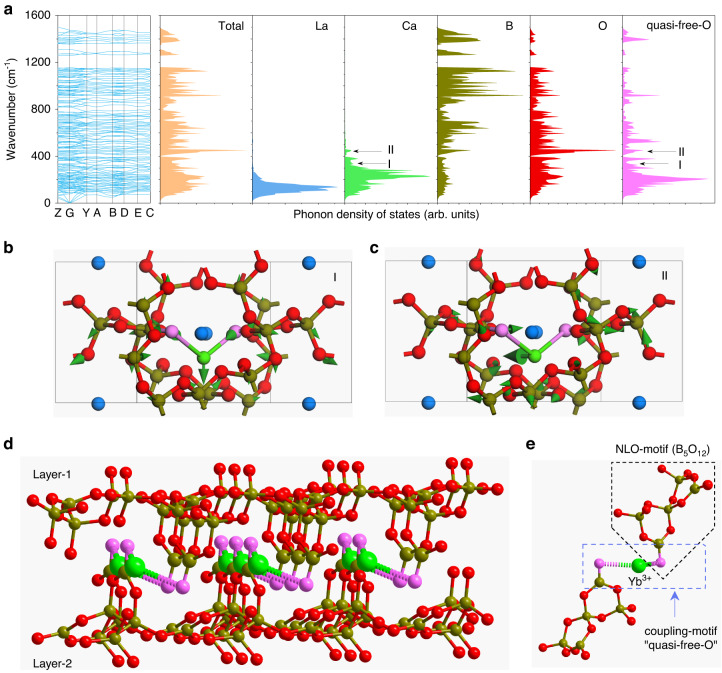
Phonon calculations of LCB crystal for lasing beyond the fluorescence spectrum. **a** Phonon dispersion and density of states of LCB crystal. **b**, **c** Lattice vibrations of “quasi-free-oxygen” at 335 cm^−1^ (mode I) and 424 cm^−1^ (mode II). The vibrational directions are labeled by green arrows. **d** The microscopic double-layer structure of Yb:LCB. Every doped Yb^3+^ ion (green balls) and two adjacent “quasi-free-oxygen” atoms (pink balls) built a functional dimer motif for electron-phonon coupling. All dimer motifs are aligned in Yb:LCB crystal. The top and bottom oxygen atoms are crystallographic equivalent. **e** The electron-phonon coupling motif (O^2-^--Yb^3+^--O^2-^) and NLO motif (B_5_O_12_) in Yb:LCB crystal

Moreover, the multiphonon-assisted mechanism of Yb:LCB crystal can be attributed to the synergistic enhancement of “quasi-free-oxygen” motif. In order to identify the distinct lattice site, we calculate the phonon dispersion and density of states of LCB crystal based on the first-principles methods. As shown in Fig. 4a, there are no imaginary modes in the phonon dispersion spectrum, suggesting LCB is kinetically stable. The partial states of La^3+^ and Ca^2+^ locate in the low-frequency region below 500 cm^−1^, and the B-O framework shows a broadband distribution from low-frequency to high-frequency. Based on the enhanced vibrational bands in Fig. 3, we deduce these two bands can be assigned to a special dangling oxygen atom in the B-O double-layer structure, namely “quasi-free-oxygen”. Differing from other oxygens linked to two boron atoms, this “quasi-free-oxygen” is only connected to one boron atom. As a result, its ligand charge is larger than others. In general, the doping Yb^3+^ can enter the La^3+^ and Ca^2+^ site simultaneously because the ionic radius of Yb^3+^ (~0.858 Å) is smaller than La^3+^ (~1.061 Å) and Ca^2+^ (~0.99 Å). However, only one zero-phonon line at 976 nm is observed in the low-temperature fluorescence spectrum (Supplementary Fig. 20). Here, we assign the dominated Yb^3+^-doping site to a Ca^2+^ site in Yb:LCB crystal, because the relatively small ionic radius difference of Ca^2+^ and Yb^3+^ ions. In addition, both Ca^2+^ and La^3+^ ions locate at a low-symmetry site, but every Ca^2+^ site is connected to two “quasi-free-oxygen”, which is favorable for synergetic energy transferring by the engineered phonons (Supplementary Fig. 21). Moreover, the heterovalent substitution of Ca^2+^ ion occupied by Yb^3+^ ion would induce a charge redistribution of surrounding ligand oxygen atoms, thus leading a constructive effect on the electron-phonon coupling intensity^35^.

Figure 4b, c displays the atomic vibrational directions at 335 cm^−1^ (mode **I**) and 424 cm^−1^ (mode **II**), which are mainly attributed to the longitudinal vibration and transverse vibration of Ca^2+^ ions (or doping Yb^3+^ ion) and “quasi-free-oxygen” atoms, respectively, accompanied with weak contribution from adjacent boron-oxygen bonds. In terms of structural chemistry, these “quasi-free-oxygen” atoms belong to one boron atom of (BO_3_)^3−^ group^36^. Compared to other oxygen atoms shared by (BO_3_)^3−^ and (BO_4_)^5−^ units, the ‘quasi-free-oxygen’ atoms possess a large valence charge and short RE-O bond length, thereby yielding a strengthened electron-phonon coupling intensity in every “O--Yb--O” motif^37^. Then, these motifs are aligned in the crystal lattice to realize a synergetic enhancement of the multiphonon coupling effect (Fig. 4d). Therefore, similar to “free-oxygen” in Yb:YCOB crystal, this “quasi-free-oxygen” unit in Yb:LCB is also demonstrated as an effective functional motif for strengthening phonon-assisted lasers. Some new borate laser crystals containing the “quasi-free-oxygen” unit, e.g., Yb:GdMgB_5_O_10_, Yb:La_2_SrB_10_O_19_, Yb:La_2_Na_2_B_10_O_19_, and Yb:YMgB_5_O_10_, should be potential candidates of gain medium for the electron-phonon coupled lasers beyond the inherent fluorescence spectrum^38,39^. In both “free-oxygen” and “quasi-free-oxygen” motif, ligand charge and RE-O bond length are two dominant factors to strengthen electron-phonon coupling intensity. This strategy can be applied to “free-nitrogen”, “free-fluorine” motifs in other inorganic solid-state materials.

## Discussion

We demonstrate the multiphonon-coupling effect and realize the extended laser wavelengths beyond the inherent fluorescence spectrum in Yb:LCB crystal. A broadband lasing emission spectrum (1000–1280 nm) is obtained by amplifying the weak multiphonon-assisted transitions step-by-step with increasing phonon numbers *n* = 1–6. Some longer laser wavelengths can be expected if coupled to more phonons (e.g., *n* > 10). However, those multiphonon-assisted transitions would suffer from the extremely weak fluorescence lineshape function, which makes it difficult to access in laser experiments.

Compared to previously reported Yb:YCOB crystal, Yb:LCB holds a small crystal field splitting, thereby avoiding a spectral overlap between pure electronic transitions and low-order phonon-assisted transitions. Meanwhile, LCB crystal provide a new structural motif, “quasi-free-oxygen”, to enhance the electron-phonon coupling intensity. This case extends the alternative active materials in rare-earth laser crystals. Besides, Yb:LCB is a non-centrosymmetric crystal, where the “quasi-free-oxygen” atom is shared by the electron-phonon coupling motif and nonlinear optical motif (B_5_O_12_) (Fig. 4e). As a result, it is possible to make a multidisciplinary coupling between multiphonon-coupled lasing and second-harmonic generation property, thus making a self-frequency doubling laser^34^ with the extended wavelengths. For example, yellow-orange lasers are in urgent demand in laser ophthalmic surgery, but difficult to obtain by semiconductor diode or rare-earth solid-state laser technology^40^. Using our Yb:LCB crystal, it is possible to make a compact yellow laser source at 581 nm with the assistance of four-phonon coupling cases. At present, this case has only been demonstrated in Yb:YCOB crystal^41^.

In summary, this work provides a novel phonon engineering in laser operation for wavelength extension, which could be easily applicable in other laser systems, such as Ti^4+^, Cr^4+^, Tm^3+^, and Er^3+^ ions. In those transition-metal doped laser materials, the excited state absorption effect needs to be considered because it can affect the tunable laser wavelength range. The possibility of extending the wavelength of laser materials opens up new perspectives in the field of ultrashort pulse lasers, terahertz emission, and frequency-comb generation. More impressively, the amplification of the electron-phonon coupling effect in the cavity could modulate the crystal lattice themselves and trigger some intriguing physical effects, e.g., cool atom, anisotropic polariton, and phonon-assisted optical parametric oscillation.

## Materials and methods

### Fluorescence spectrum experiment

The fluorescence spectra of Yb:LCB crystal are collected by an Edinburgh fluorescence spectrometer at various temperatures (T = 120, 170, 220, 270, and 300 K). Three crystal plates with 1 mm thickness are used to avoid the reabsorption effect as much as possible. An 880 nm LD is used as the pump source. The excitation and emission slit functions are set to 4 and 1. The wavelength step is 0.5 nm and the dwell time is 0.02 s. Every spectral line is collected with two repeats.

### Laser experiment

The 10 at.% Yb^3+^-doped LCB crystals are cut along the principal axes (X, Y, Z) with dimensions of 3 × 3 × 6 mm^3^, and the two 3 mm × 3 mm faces are polished for laser experiments. The pump source is a fiber-coupled InGaAs laser with a center wavelength of 976 nm and a fiber diameter of 105 μm. The maximum output power is 30 W. The pump light was focused into crystal by a coupling system with a beam ratio of 1:1 and a focal length of 9.5 cm. The beam radius in the crystal is about 52 μm. The absorption ratio under lasing conditions is about 41%. To reduce the thermal effect of the crystal in the experiment process, Yb:LCB crystal was wrapped with indium foil and mounted in a water-cooled copper block. A filter mirror with HR-coated at 976 nm is placed behind the output coupler to reflect the residual pump light. The laser output power is collected by a power meter (Newport, Model 1916-R), and the laser spectrum is recorded by two different spectrometers, wavelength λ < 1140 nm (Ocean Optics, HR4000, spectrometer resolution 1 nm), and wavelength λ > 1140 nm (A.P.E.--WaveScan, S/N S09668, spectrometer resolution 0.2 nm).

We use a simple monolithic resonant cavity in laser experiments. The schematic diagram was plotted in Supplementary Fig. 4.

1. phonon-assisted laser with phonon number *n* = 1
  A plano-concave cavity is utilized with input mirror M1 and output coupler M2. The coatings on the mirrors are designed as M1 is coated with high-transmission (HT, T > 99%) at 976 nm and high-reflection (HR) at 1000–1100 nm, and M2 is HR-coated at 1000–1100 nm (T = 0.2%). The total cavity length is around 97 mm. A quartz birefringence filter with a thickness of 1 mm is inserted along the Brewster’s angle to tune the laser wavelength.
2. multiphonon-assisted laser with phonon number *n* = 2
  A plano-concave cavity is utilized with input mirror M1 and output coupler M2. The coatings on the mirrors are designed as M1 is coated with high-transmission (HT, T > 99%) at 976 nm and high-reflection (HR) at 1000–1100 nm, M2 is HT-coated at 1000–1040 nm (T > 90%), and HR-coated at 1080–1200 nm. The transmittance of the output coupler (T_oc_) around 1050 nm is 5%. The total cavity length is around 100 mm.
3. multiphonon-assisted laser with phonon number *n* = 3
  A short plano-plano cavity is utilized and the input and output mirrors are directly coated on the front and end faces of Yb:LCB crystal. The coatings on the mirrors are designed as M1 is coated with high-transmission (HT, T > 99%) at 970–1060 nm and high-reflection (HR) at 1080–1200 nm, M2 is HT-coated at 1000–1060 nm (T > 90%) and HR-coated at 1080–1200 nm (T = 0.1%).
4. multiphonon-assisted laser with phonon number *n* = 4
  A short plano-plano cavity is utilized and the input and output mirrors are directly coated on the front and end faces of Yb:LCB crystal. The coatings on the mirrors are designed as M1 is coated with high-transmission (HT, T > 99%) at 970–1120 nm and high-reflection (HR) at 1140–1200 nm, M2 is HT-coated at 1000–1120 nm (T > 90%) and HR-coated at 1140–1200 nm (T = 0.1%).
5. multiphonon-assisted laser with phonon number *n* = 5
  A short plano-plano cavity is utilized and the input and output mirrors are directly coated on the front and end faces of Yb:LCB crystal. The coatings on the mirrors are designed as M1 is coated with high-transmission (HT, T > 99%) at 970–1190 nm and high-reflection (HR) at 1210–1300 nm, M2 is HT-coated at 1020–1190 nm (T > 90%) and HR-coated at 1210–1300 nm (T = 0.1%).
6. multiphonon-assisted laser with phonon number *n* = 6

A short plano-plano cavity is utilized and the input and output mirrors are directly coated on the front and end faces of Yb:LCB crystal. The coatings on the mirrors are designed as M1 is coated with high-transmission (HT, T > 99%) at 970–1240 nm and high-reflection (HR) at 1260–1500 nm, M2 is HT-coated at 1020–1240 nm (T > 90%) and HR-coated at 1260–1500 nm (T = 0.1%).

In addition, we measured the fundamental characteristics of multiphonon-assisted Yb:LCB laser beyond the fluorescence spectrum. The oscilloscope trace exhibits that the multiphonon-assisted laser is a continuous-wave laser (Supplementary Fig. 8). The full widths at half maximum of the laser linewidths are smaller than 0.2 nm around 1162 nm, which is consistent with the feature of the continuous-wave laser emission of Yb^3+^ ions (Supplementary Fig. 9). The beam quality factor ${{\rm{M}}}_{{\rm{x}}}^{2}$ and ${{\rm{M}}}_{{\rm{y}}}^{2}$ are 1.38 and 1.42 at 1107 nm, 1.39 and 1.45 at 1162 nm, 1.27 and 1.55 at 1235 nm, along the x-axis and y-axis respectively (Supplementary Fig. 10). In addition, power stability is very important for practical applications. As shown in Supplementary Fig. 11, the power curve of temporal stability was measured continuously over a period of 30 min. The power fluctuation at 1107 nm is less than 3%, and the power fluctuation at 1162 nm is less than 5%, thus indicating these multiphonon-assisted lasers beyond the fluorescence spectrum are very stable. Moreover, we use a Glan-Taylor polarizer to detect laser polarization. Supplementary Fig. 12 shows that the laser beyond the fluorescence spectrum is linearly polarized. The polarization direction at 1107, 1162, and 1235 nm is E//X in Y-cut LCB crystal. Finally, we also realize a similar lasing outside the fluorescence spectrum in X-cut and Z-cut Yb:LCB crystal (Supplementary Fig. 13), indicating multiphonon coupling effect is also applicable in other two-principle axis directions. In comparison, the laser performance of Y-cut crystal is the best one. In addition, we modified the Yb concentration with 5 at.% and 10 at.%. We find 10 at.% Yb:LCB crystal exhibits better laser performances with low threshold and high slope efficiency (Supplementary Fig. 14). Therefore, we used 10 at.% Yb-doped LCB crystals in laser experiments.

In order to affirm our lasing beyond the fluorescence spectrum, we exclude the possible influence of the high temperature and coating film for the fluorescence spectrum. There is no additional fluorescence signal at 1100–1200 nm under high temperatures up to 250 °C (Supplementary Fig. 15), thereby indicating this lasing beyond the fluorescence spectrum is indeed devoted to electron-phonon coupling effect, but not thermal-induced spectral broadening. There is also no additional fluorescence signal at 1100–1200 nm in a coated Yb:LCB crystal (Supplementary Fig. 16), thus excluding the possible fluorescence emission from coating films.

### Tunable laser experiment

In our tunable laser experiment, a plano-concave cavity is utilized with input mirror M1 and output coupler M2. The coatings on the mirrors are designed as follows: M1 is coated with high-transmission (HT, T > 99%) at 976 nm and high-reflection (HR) at 995–1100 nm, M2 is partially transmittance at 995–1100 nm (T_oc_ = 0.2%). A quartz birefringent filter (thickness d = 1 mm) is inserted along Brewster’s angle. By rotating the BF, a continuously tunable laser from 996 to 1051 nm can be obtained (Supplementary Fig. 17). There are two peaks around 1000 and 1034 nm on the tunable power curve with a maximum output power of 153 mW and 1034 nm. We also tried the tunable laser generation with high phonon numbers involved. A wavelength shift from 1086 to 1113 nm was observed in Yb:LCB crystal (Supplementary Fig. 18).

### Supplementary information

Supporting Information

## References

[1] Bardeen J, Cooper LN, Schrieffer JR (1957). Theory of superconductivity. Phys. Rev. J. Arch..

[2] Chen ZY (2021). Anomalously strong near-neighbor attraction in doped 1D cuprate chains. Science.

[3] Pearson RG (1975). Concerning jahn-teller effects. Proc. Natl Acad. Sci. USA.

[4] Jin WC (2020). Observation of the polaronic character of excitons in a two-dimensional semiconducting magnet CrI_3_. Nat. Commun..

[5] Paradisanos I (2021). Efficient phonon cascades in WSe_2_ monolayers. Nat. Commun..

[6] Dai YC (2023). Phonon-assisted upconversion in twisted two-dimensional semiconductors. Light Sci. Appl..

[7] Wang BY (2022). Electron-phonon coupling-assisted universal red luminescence of o-phenylenediamine-based carbon dots. Light Sci. Appl..

[8] Jadczak J (2019). Room temperature multi-phonon upconversion photoluminescence in monolayer semiconductor WS_2_. Nat. Commun..

[9] Liang F (2022). Multiphonon-assisted lasing beyond the fluorescence spectrum. Nat. Phys..

[10] Johnson LF, Dietz RE, Guggenheim HJ (1963). Optical maser oscillation from Ni^2+^ in MgF_2_ involving simultaneous emission of phonons. Phys. Rev. Lett..

[11] Suzuki A, Kränkel C, Tokurakawa M (2020). High quality-factor Kerr-lens mode-locked Tm: Sc_2_O_3_ single crystal laser with anomalous spectral broadening. Appl. Phys. Express.

[12] Zhao YG (2021). Kerr-lens mode-locked Tm-doped sesquioxide ceramic laser. Opt. Lett..

[13] Kifle E (2020). Watt-level diode-pumped thulium lasers around 2.3 µm. Appl. Opt..

[14] Loiko P (2016). Vibronic thulium laser at 2131 nm *Q*-switched by single-walled carbon nanotubes. J. Opt. Soc. Am. B.

[15] Moulton PF (1986). Spectroscopic and laser characteristics of Ti: Al_2_O_3_. J. Opt. Soc. Am. B.

[16] Walling J (1980). Tunable alexandrite lasers. IEEE J. Quantum Electron..

[17] Sennaroglu A (2002). Broadly tunable Cr^4+^-doped solid-state lasers in the near infrared and visible. Prog. Quantum Electron..

[18] Chen P (2022). 12.3-W output power and 271-nm wavelength tunability of diode-double-end-pumped Tm: CALYO laser. Opt. Laser Technol..

[19] Nakamura S (2009). Broadly tunable Yb^3+^-doped Y_3_Al_5_O_12_ ceramic laser at room temperature. Jpn. J. Appl. Phys..

[20] Loiko, P. et al. Multiphonon-assisted emission of rare-earth ions: towards pulse shortening in mode-locked lasers. In *Optica Advanced Photonics Congress 2022* (Optica, 2022).

[21] Kimura S, Tani S, Kobayashi Y (2019). Raman-assisted broadband mode-locked laser. Sci. Rep..

[22] Jiang Y (2020). Organic solid-state lasers: a materials view and future development. Chem. Soc. Rev..

[23] Jiang Y (2021). Frequency-upconverted stimulated emission by up to six-photon excitation from highly extended spiro-fused ladder-type oligo(*p*-phenylene)s. Angew. Chem. Int. Ed..

[24] Toncelli A (2022). Light in the darkness. Nat. Phys..

[25] Wang FY (2022). Anion-centered polyhedron strategy for strengthening photon emission induced by electron-phonon coupling. Inorg. Chem..

[26] Brenier A (2012). Lasing Yb^3+^ in crystals with a wavelength dependence anisotropy displayed from La_2_CaB_10_O_19_. Appl. Phys. B.

[27] Guo R (2005). Growth and spectroscopic properties of ytterbium-doped lanthanum calcium borate (Yb^3+^: La_2_CaB_10_O_19_) crystal. Opt. Commun..

[28] Wang SL (2013). Efficient Ho: LuLiF_4_ laser diode-pumped at 1.15 μm. Opt. Express.

[29] Paschotta R (1997). 230 mW of blue light from a thulium-doped upconversion fiber laser. IEEE J. Sel. Top. Quantum Electron..

[30] Saki N (2017). Picosecond laser applications in aesthetic dermatology. J. Surg. Dermatol..

[31] Huang K, Rhys A (1950). Theory of light absorption and non-radiative transitions in F-centres. Proc. R. Soc. A Math. Phys. Eng. Sci..

[32] Riseberg LA, Moos HW (1968). Multiphonon orbit-lattice relaxation of excited states of rare-earth ions in crystals. Phys. Rev. J. Arch..

[33] Huang K, Gu ZQ (1982). Phonon analysis in multiphonon transitions. Commun. Theor. Phys..

[34] Cheng YL (2022). Diode-pumped continuous-wave laser of a self-frequency-doubling Yb:La_2_CaB_10_O_19_ crystal. Opt. Lett..

[35] Blasse G (1990). Interaction between optical centers and their surroundings: an inorganic chemist’s approach. Adv. Inorg. Chem..

[36] Wu YC (2001). A new lanthanum and calcium borate La_2_CaB_10_O_19_. Chem. Mater..

[37] Cheng YL (2022). Enhanced electron-phonon coupling effect in rare-earth borate crystals containing a "quasi-free-oxygen" motif. Inorg. Chem..

[38] Huang YS (2017). Spectroscopy and laser performance of Yb^3+^:GdMgB_5_O_10_ crystal. J. Lumin..

[39] You ZY (2017). Simultaneous Q-switched orthogonally polarized dual-wavelength Yb^3+^:GdMgB_5_O_10_ laser. Opt. Mater. Express.

[40] Thoss A (2019). New diode-pumped solid-state laser emits in the yellow region. Laser Focus World. Mag. Photonics Optoelectron. Ind..

[41] Fang Q (2016). Self-frequency-doubled vibronic yellow Yb: YCOB laser at the wavelength of 570 nm. Opt. Lett..

