# Supplementary material for: Phonon engineering in Yb:La2CaB10O19 crystal for extended lasing beyond the fluorescence spectrum
Source: Light Sci Appl. 2023 Aug 25;12:203. doi: 10.1038/s41377-023-01243-x (PMC10457375; doi:10.1038/s41377-023-01243-x)
Supplement: Supplementary file 1 — Supporting Information [file 41377_2023_1243_MOESM1_ESM.docx]

**Supporting Information for**

**Phonon engineering in Yb:La_2_CaB_10_O_19_ crystal for extended lasing beyond the fluorescence spectrum**

Yanling Cheng,^1^ Fei Liang,^1,*^ Dazhi Lu,^1^ Jingcheng Feng,^2^ Guochun Zhang,^2^ Haohai Yu,^1,*^ Huaijin Zhang,^1,*^ Yicheng Wu^1^

^1^ State Key Laboratory of Crystal Materials and Institute of Crystal Materials, Shandong University, Jinan 250100, China

^2^ Key Lab Functional Crystals and Laser Technology, Technical Institute of Physics and Chemistry, Chinese Academy of Sciences, Beijing 100190, China

Corresponding authors

**Email:**

* liangfei@sdu.edu.cn (F. Liang)

* haohaiyu@sdu.edu.cn (H.H. Yu)

* huaijinzhang@sdu.edu.cn (H.J. Zhang)

**Additional figures and tables**

**
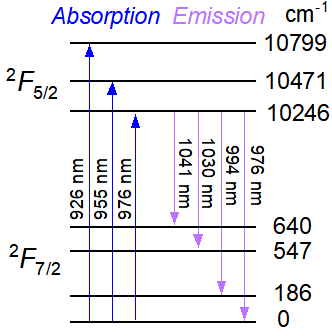
**

**Supplementary Fig. 1 |** The energy splitting of Yb:LCB crystal.


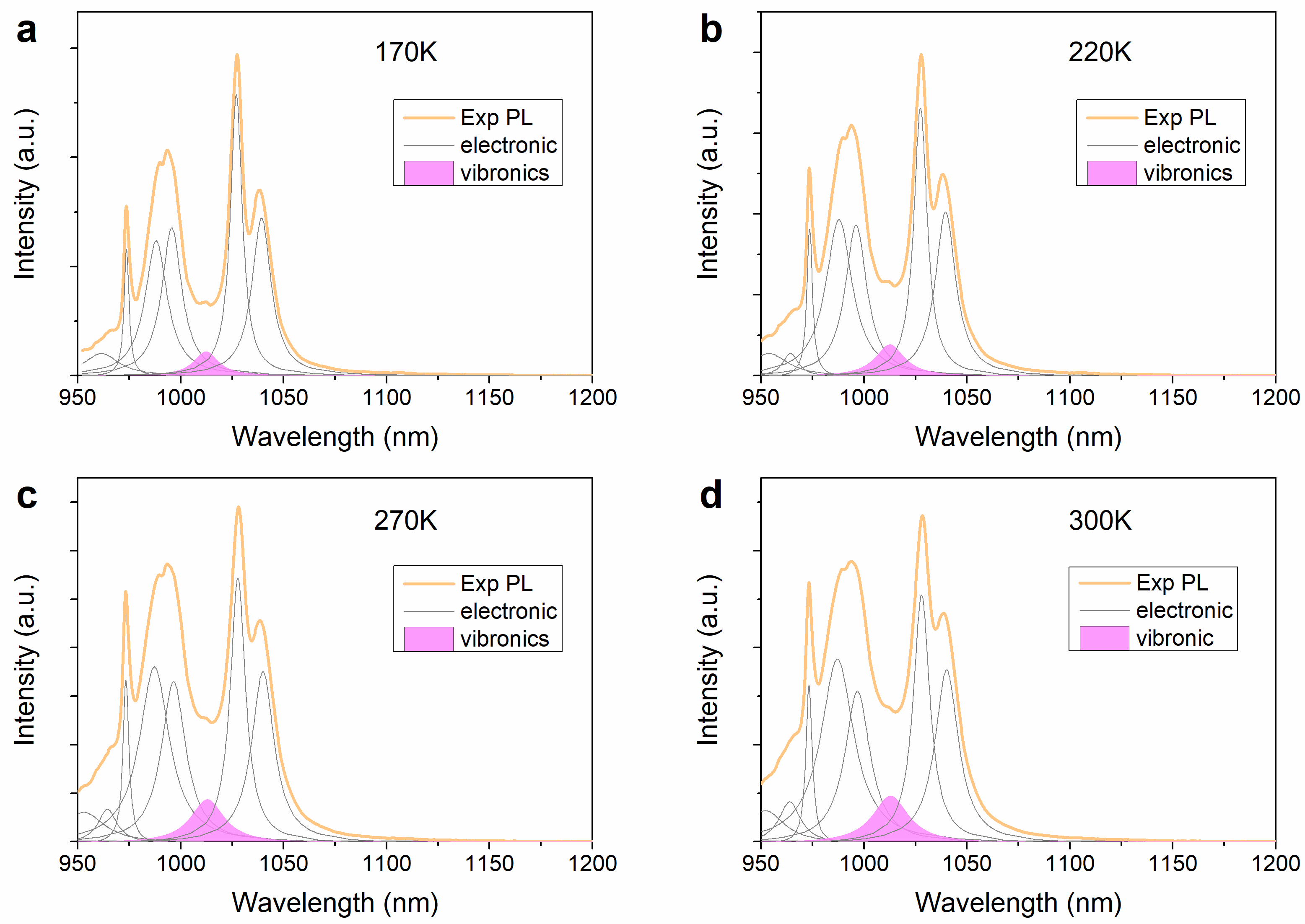


**Supplementary Fig. 2 | The peaks fitting of fluorescence spectrum for Yb:LCB crystal.** (a) T=170 K, (b) T=220 K, (c) T=270 K, (d) T=300 K. The pink region represents phonon-assisted fluorescence emission.


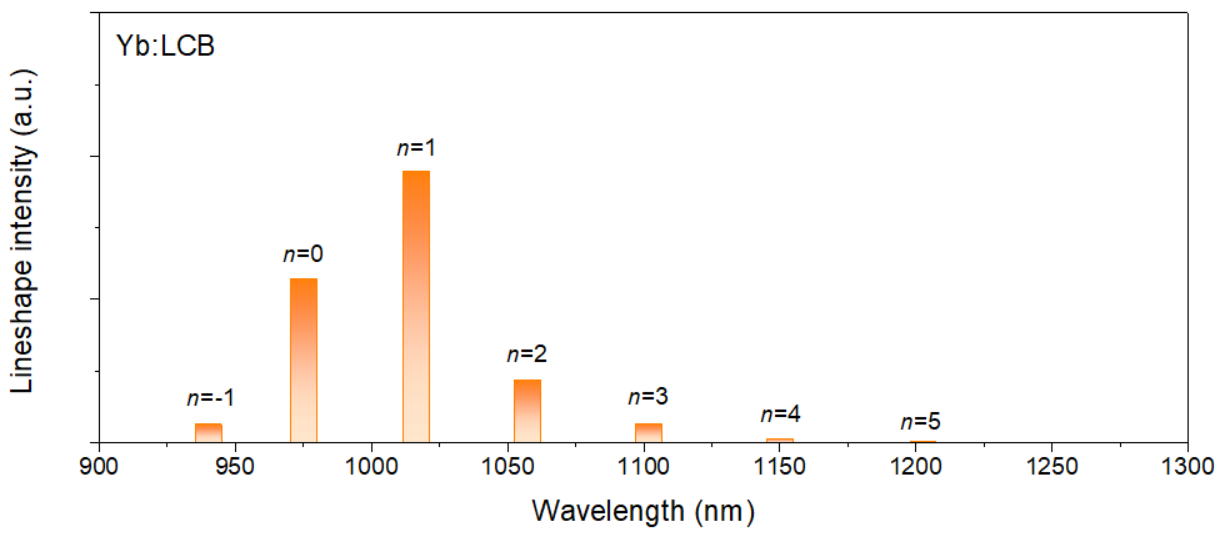


**Supplementary Fig. 3 |** Calculated fluorescence lineshape of multiphonon-assisted transitions in Yb:LCB crystal (T = 300 K, S = 1.02). *n* represents the phonon number. The fluorescence lineshape dramatically decrease when the coupled phonon number n>1. Therefore, in practical laser experiments, it still a great challenge to realize high-order phonon coupled lasing (n>10) owing to its extremely weak lineshape, because all low-phonon ones need to be suppressed. It can be deduced that the lasing threshold would be very high (even beyond the intrinsic damage threshold of laser crystal). At present, we obtained the longest lasing wavelength around 1280 nm with involved phonon number *n*=6. In the future, we will try longer wavelengths with optimized resonant cavity.


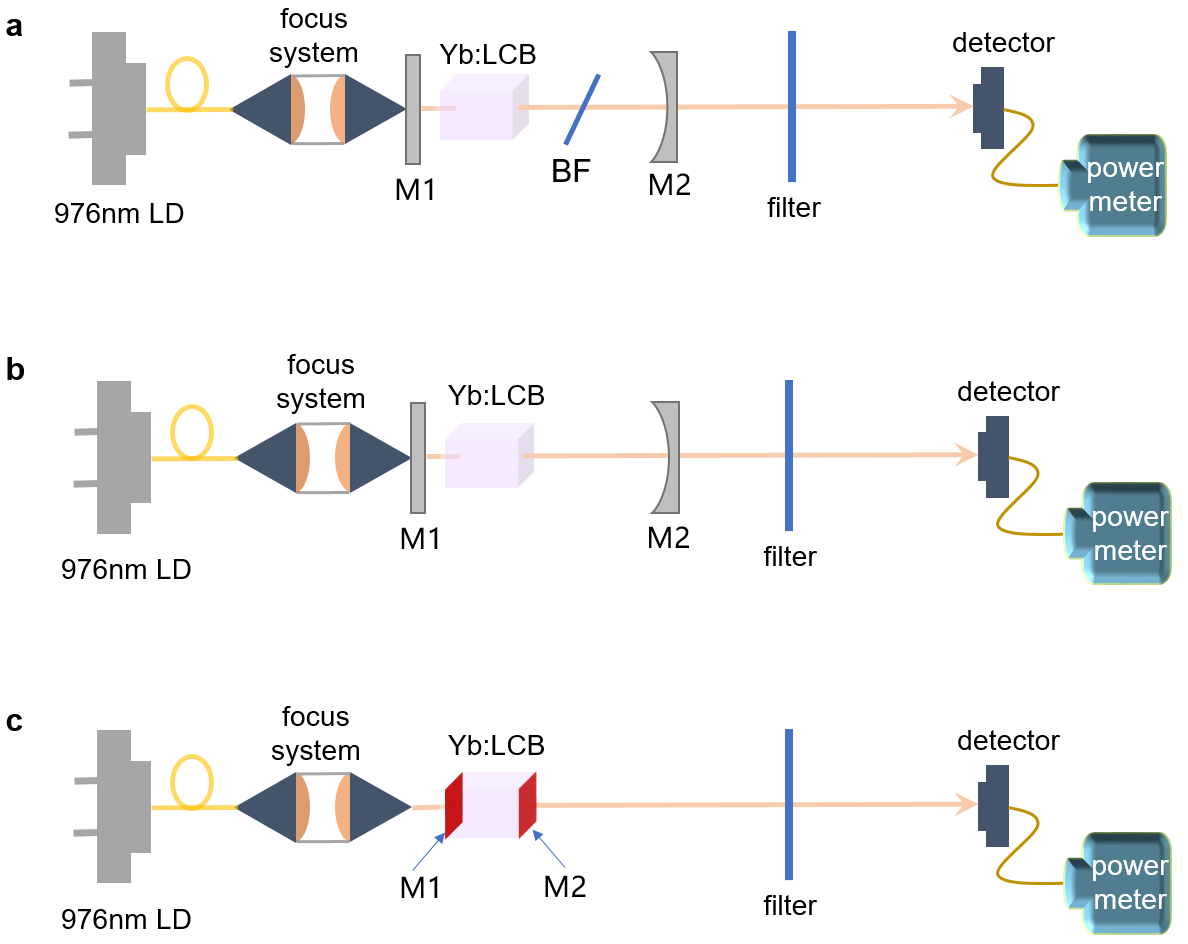

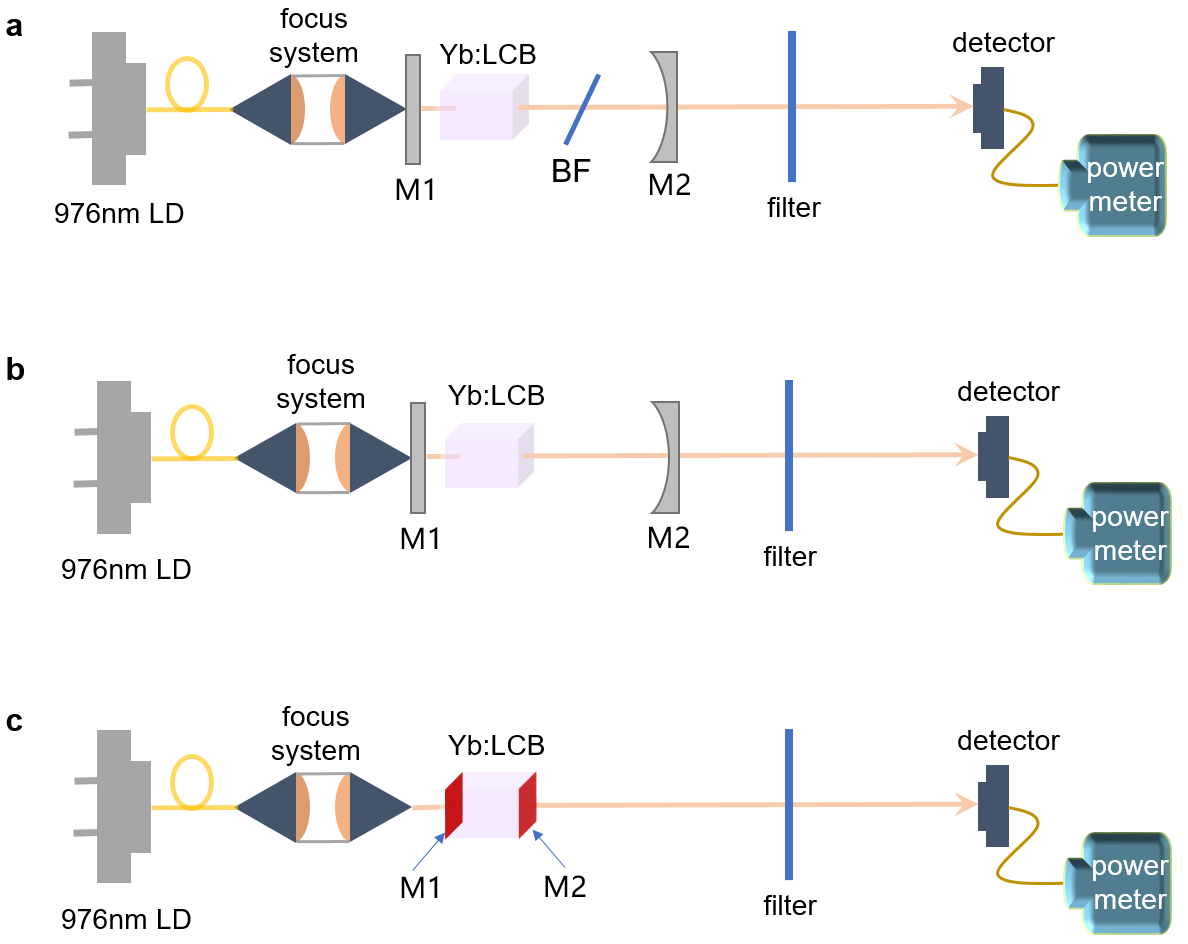

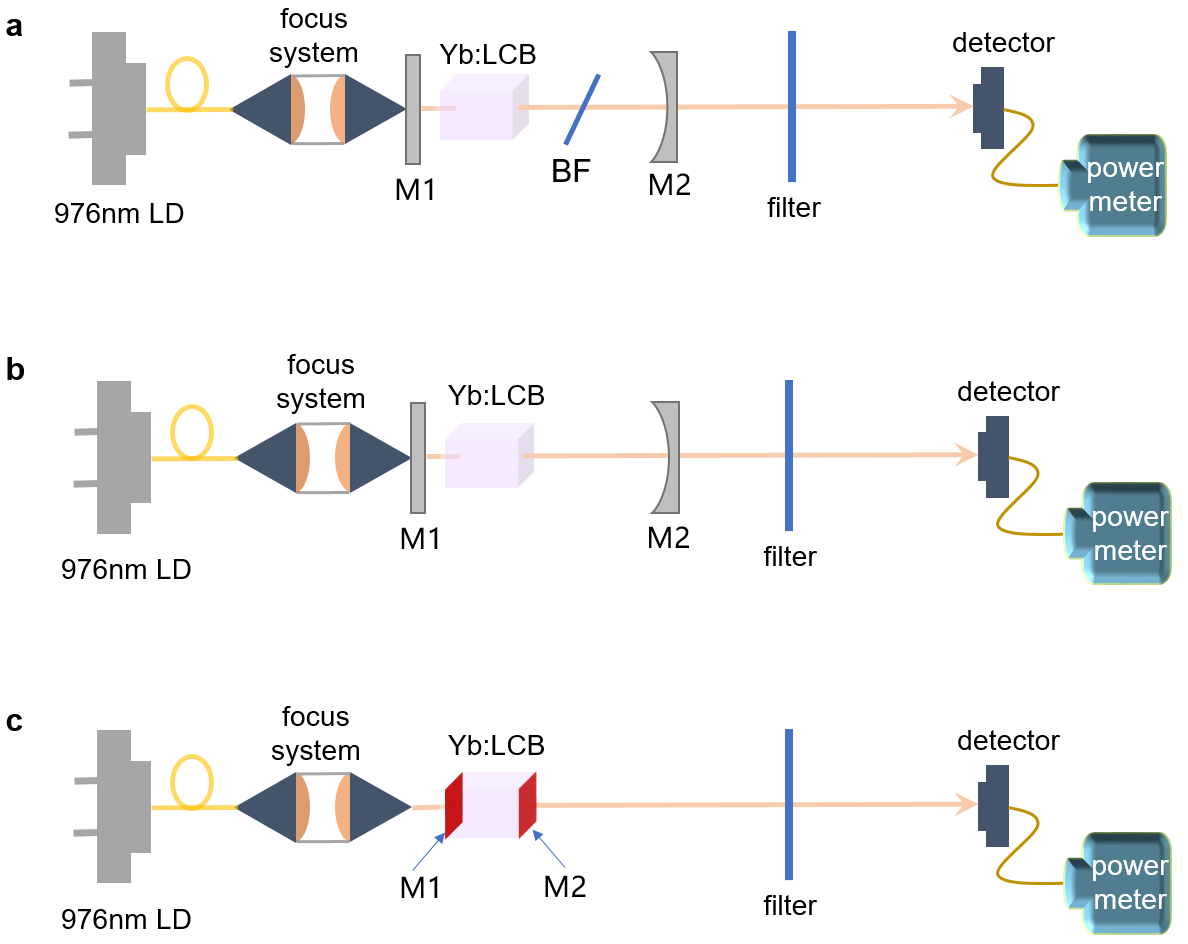


**Supplementary Fig. 4 |** The laser experimental setup. (a) λ = 1006 nm, (b) λ = 1051 nm, (c) λ = 1107, 1160, and 1235 nm.


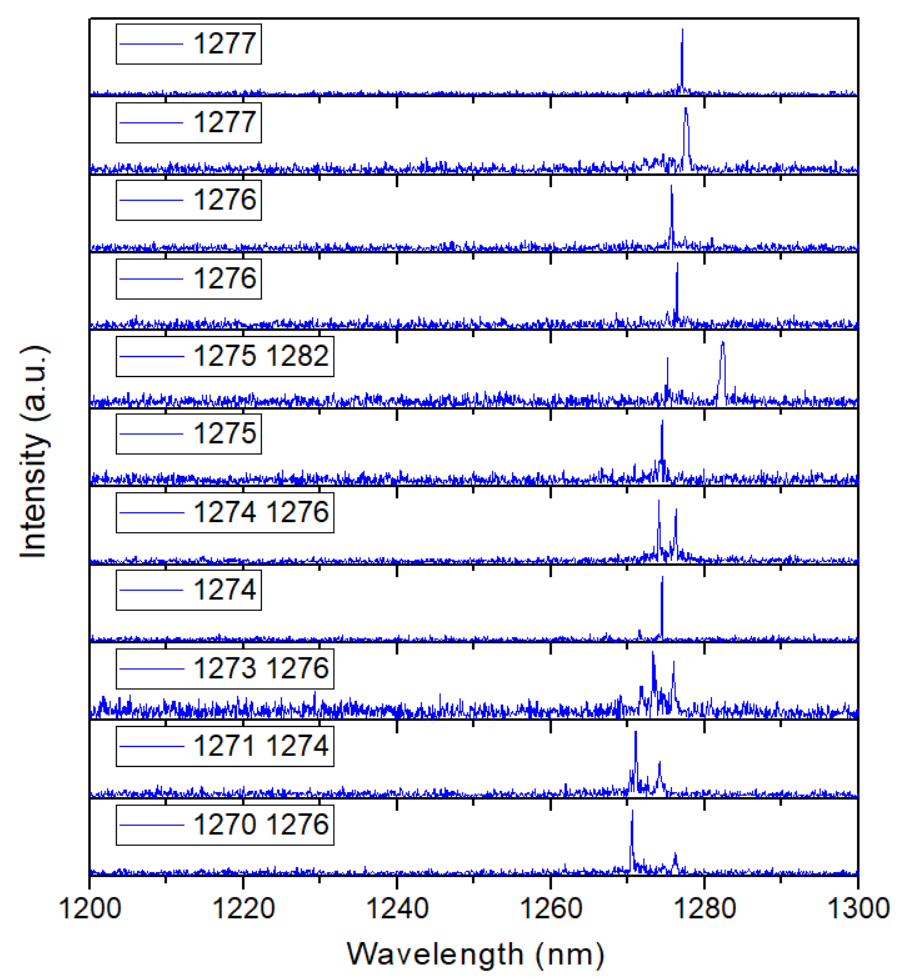


**Supplementary Fig. 5 |** Laser spectrum around 1270-1280 nm in Yb:LCB crystal. The output power is very low (< 1 mW).


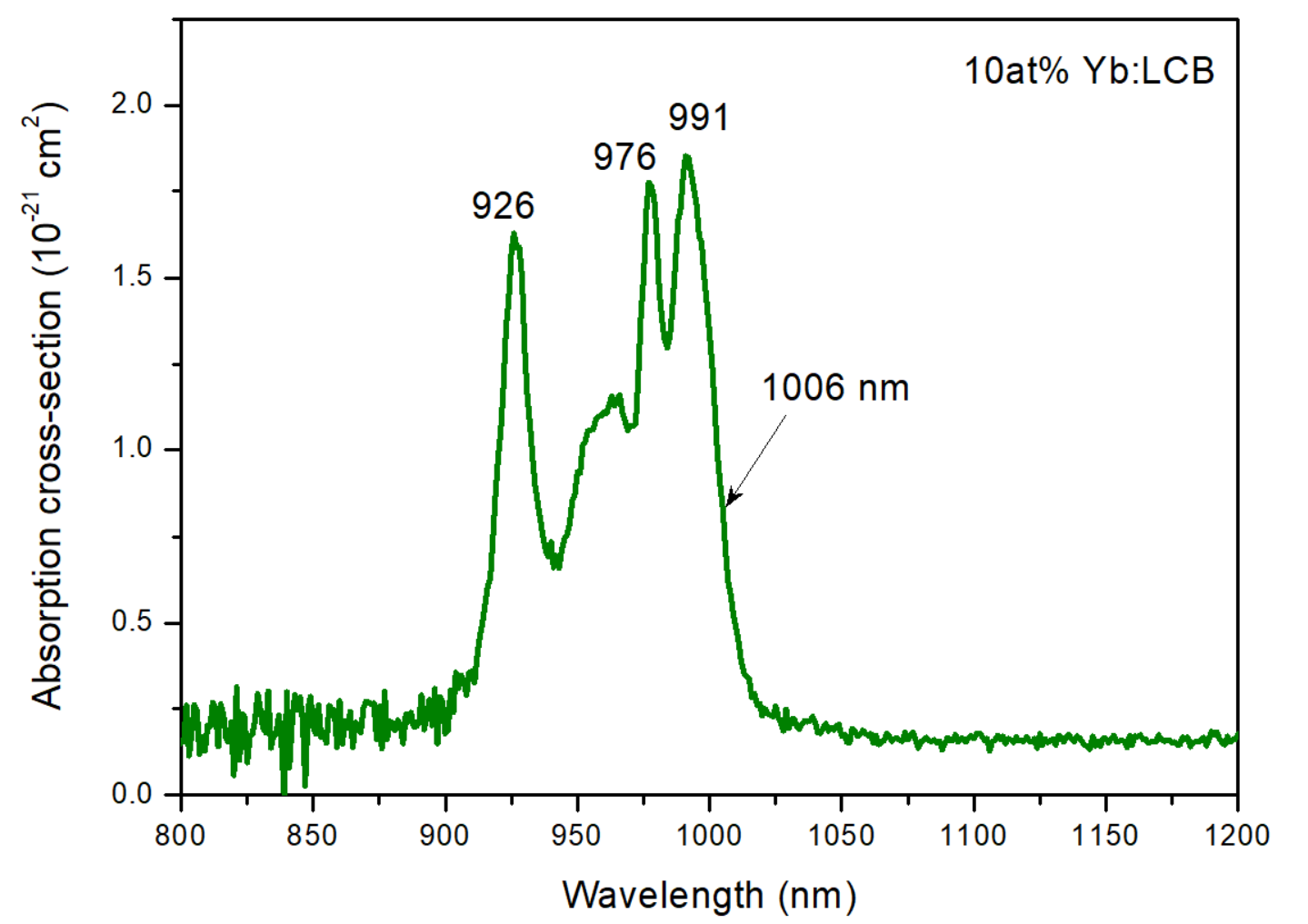


**Supplementary Fig. 6 |** The absorption cross-section of Yb:LCB crystal.


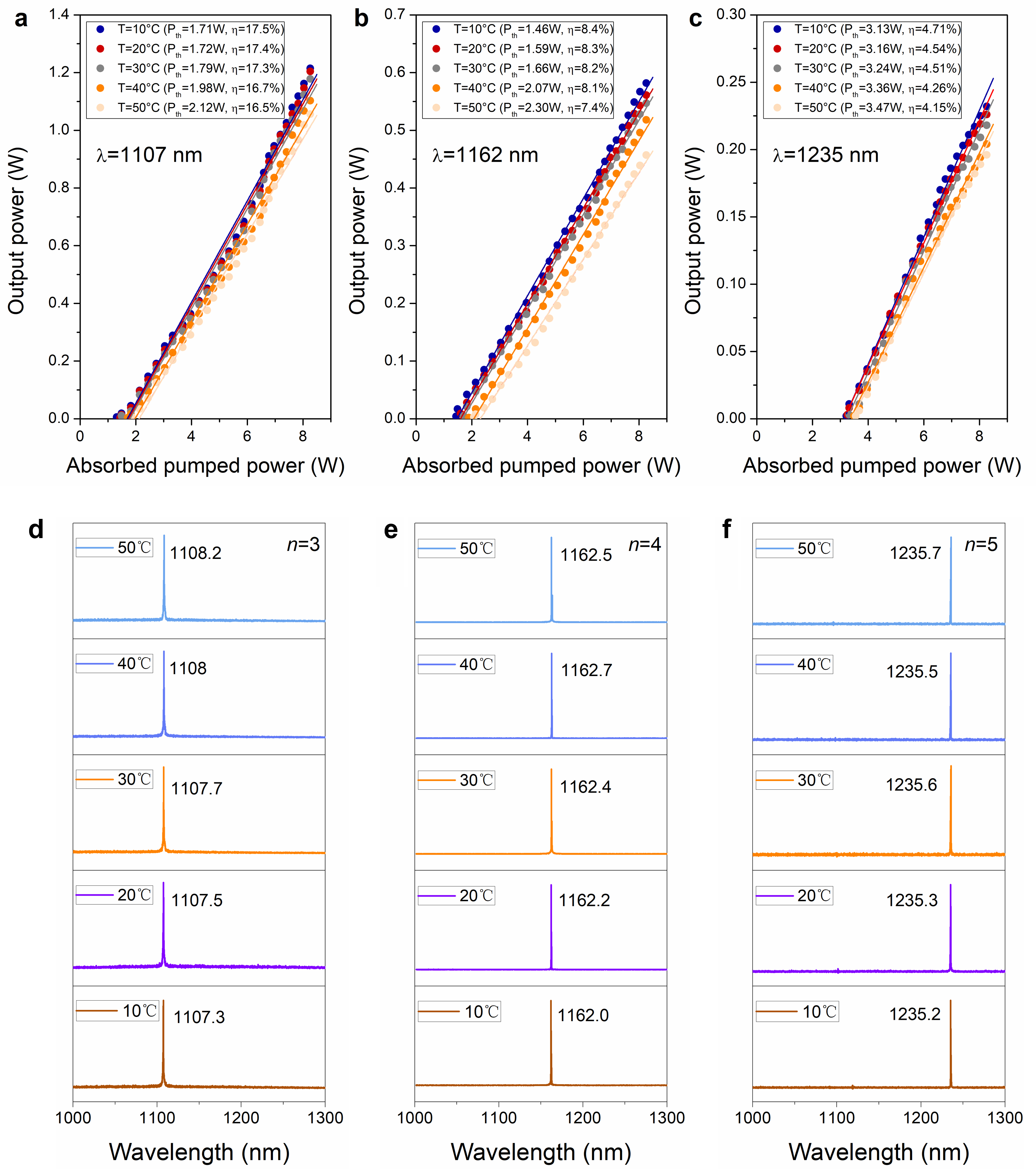


**Supplementary Fig. 7 |** The temperature dependent of laser performances. Output power at (a) 1107 nm, (b) 1162 nm, and (c) 1235 nm. The laser performance slightly deteriorates with increasing temperatures. These results indicate that the quasi-three-level Yb^3+^-lasers also exhibits a strong temperature dependence at the wavelength outside the fluorescence spectrum. The temperature dependent of laser wavelengths at (d) 1107 nm, (e) 1162 nm, and (f) 1235 nm. The laser wavelengths exhibit a slightly shift under different temperatures. This small frequency shift can be attributed to two aspects. One is the zero-phonon line (ZPL) redshift with the increasing temperature owing to the reduced ^2^F_5/2_-^2^F_7/2_ energy level difference induced by L-S coupling. Another is the laser wavelength blueshift owing to the reduced phonon frequency induced by lattice thermal expansion of the Yb:LCB crystal. The final laser frequency shift is a synergistic effect of both two factors.

**
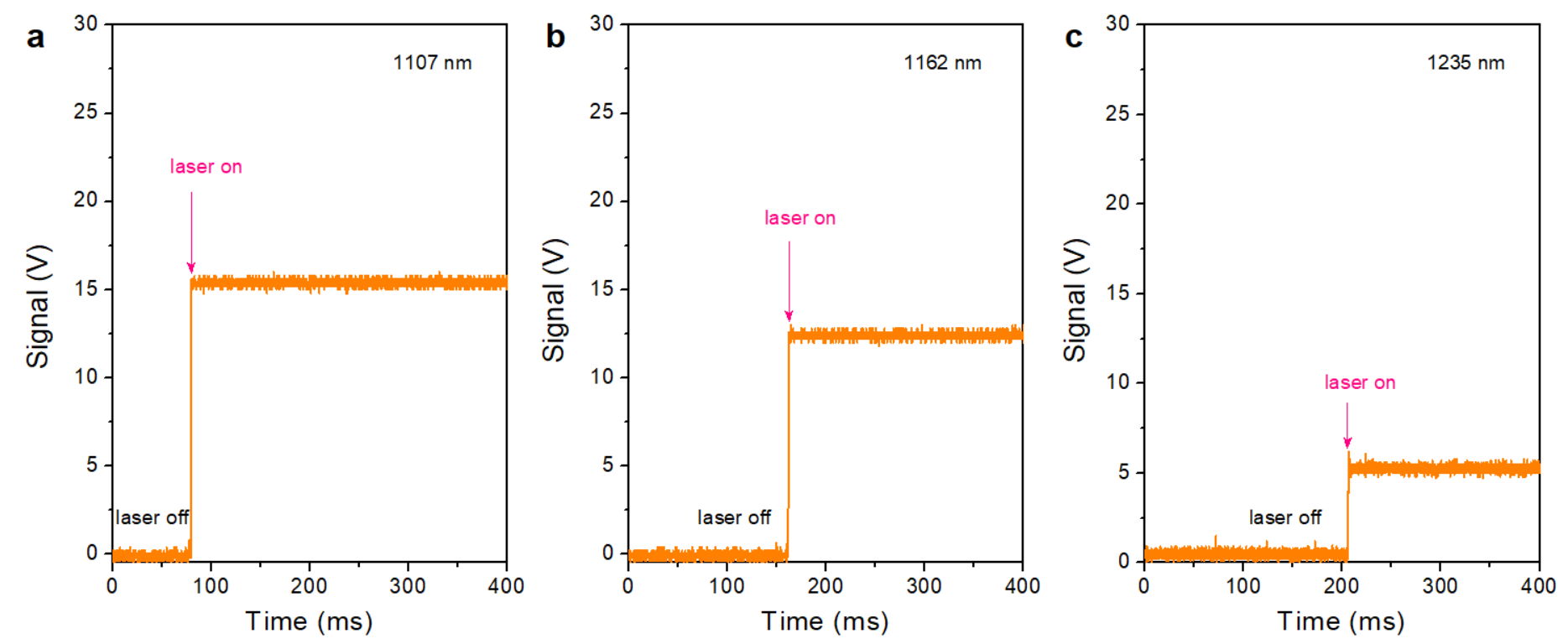
**

**Supplementary Fig. 8 |** Typical oscilloscope trace of laser emission at (a) 1107 nm, (b) 1162 nm and (c) 1235 nm in Y-cut Yb:LCB crystal.


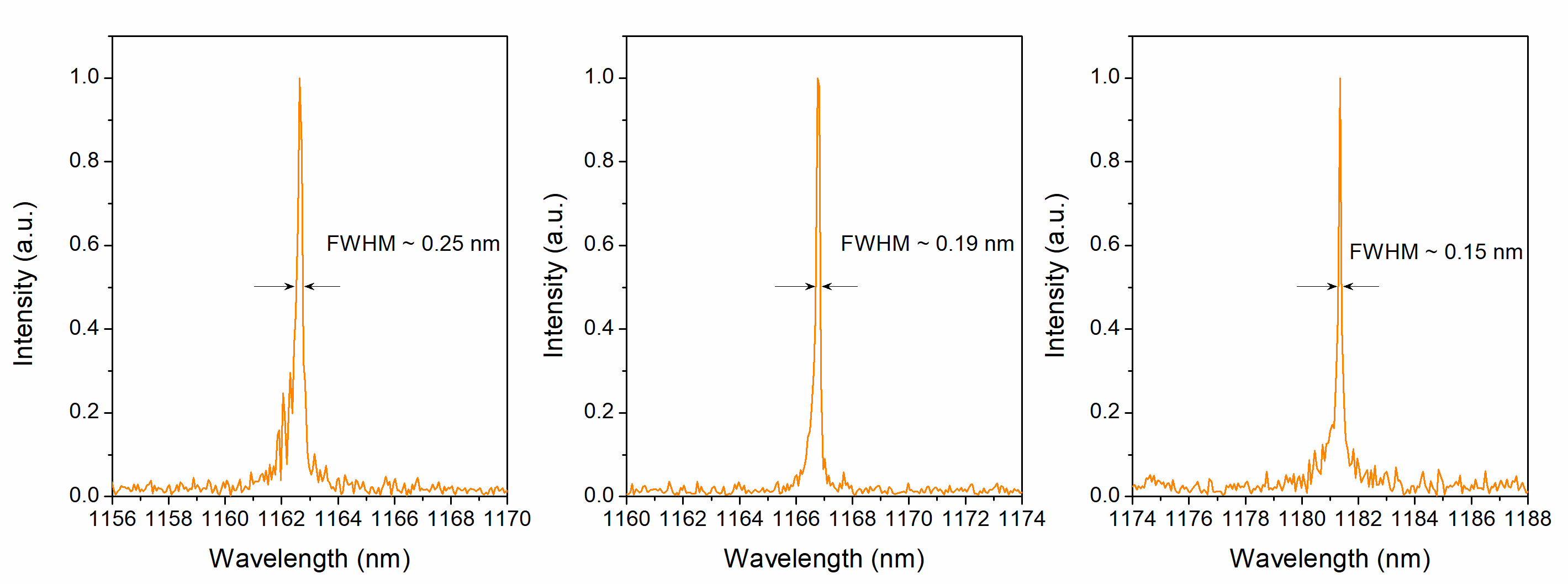


**Supplementary Fig. 9 |** The laser linewidth around 1160 - 1180 nm. The resolution of the spectrometer for spectral measurement is 0.05 nm (Fourier Transform Optical Spectrum Analyzer, Thorlabs, OSA205C).


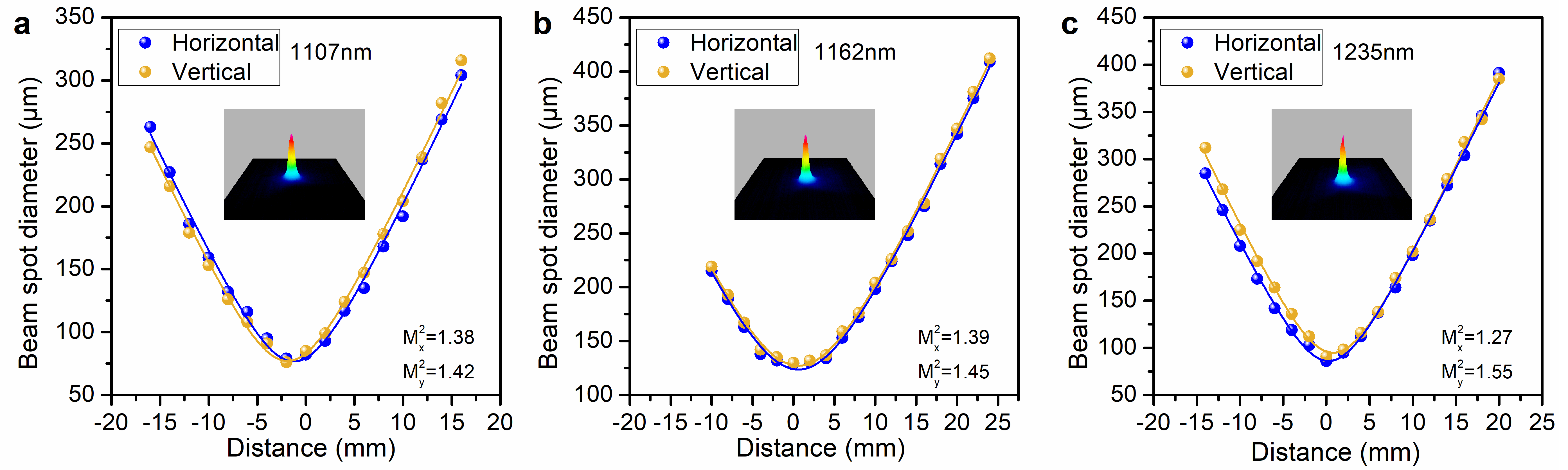


**Supplementary Fig. 10 |** **The beam quality factor of multiphonon-assisted lasers.** (a) 1107 nm, (b) 1162 nm, (c) 1235 nm in Y-cut Yb:LCB crystal.


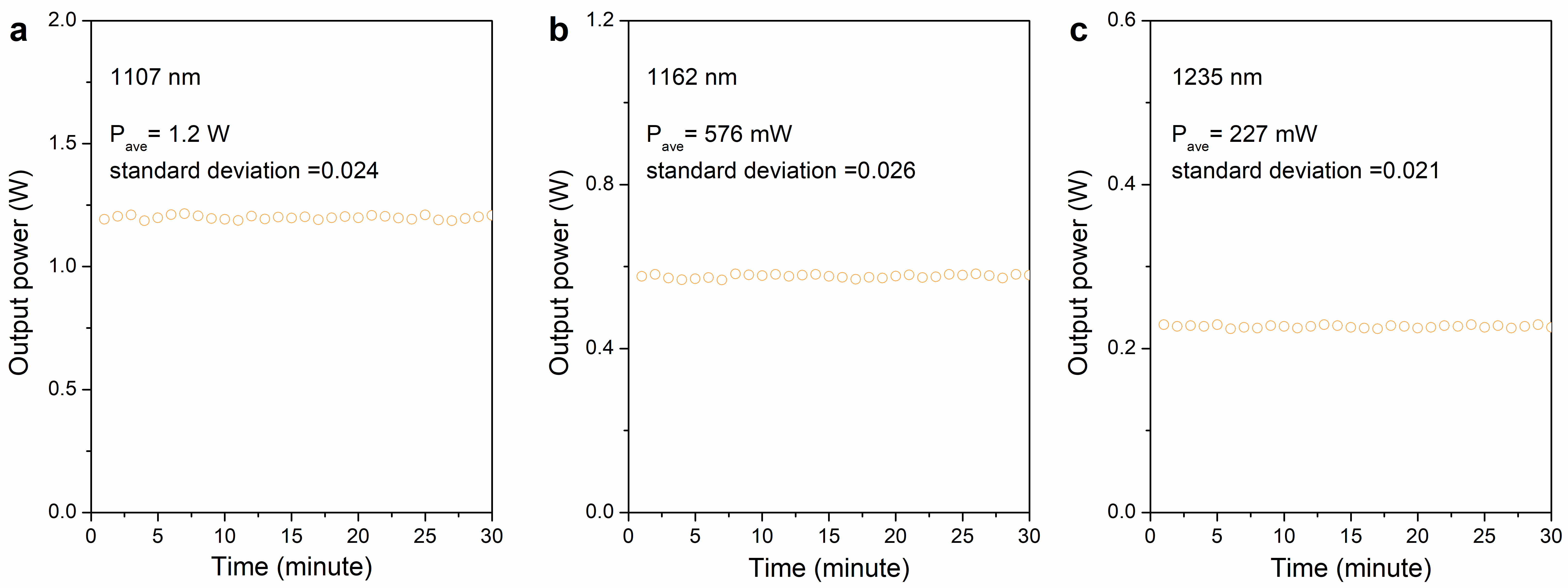


**Supplementary Fig. 11 |** **Power curve of temporal stability of multiphonon-assisted lasers.** (a) 1107 nm, (b) 1162 nm, (c) 1235 nm in Y-cut Yb:LCB crystal.


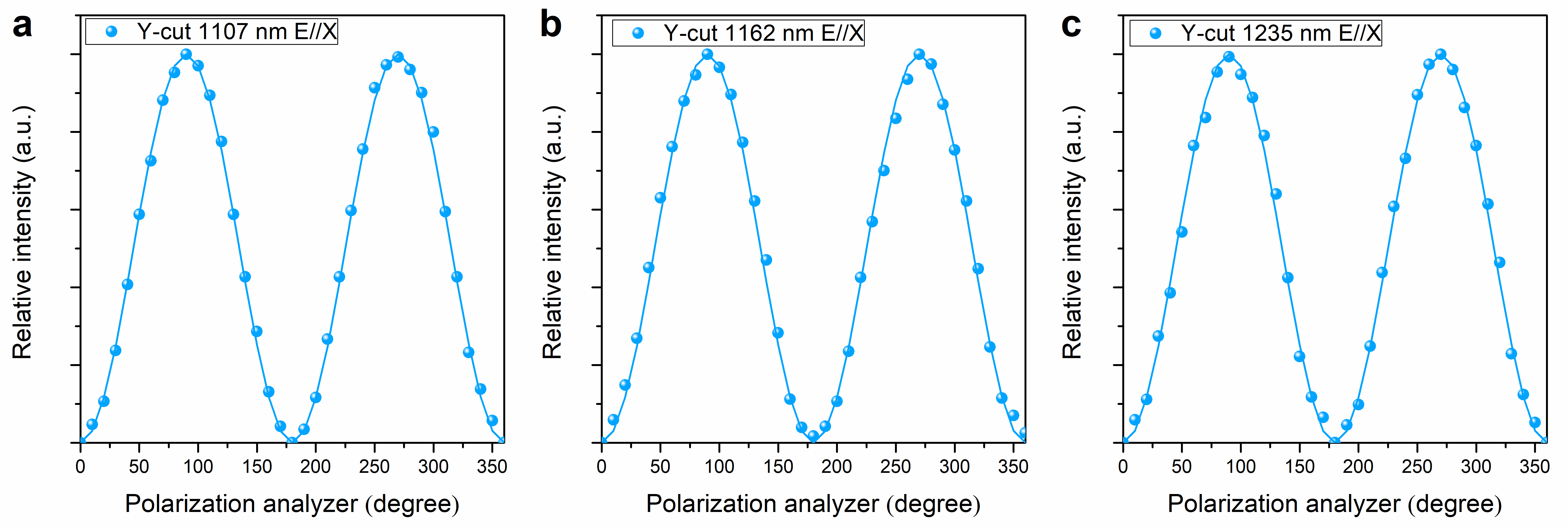


**Supplementary Fig. 12 |** **Laser polarization measurement of multiphonon-assisted lasers.** (a) 1107 nm, (b) 1162 nm, (c) 1235 nm in Y-cut Yb:LCB crystal.


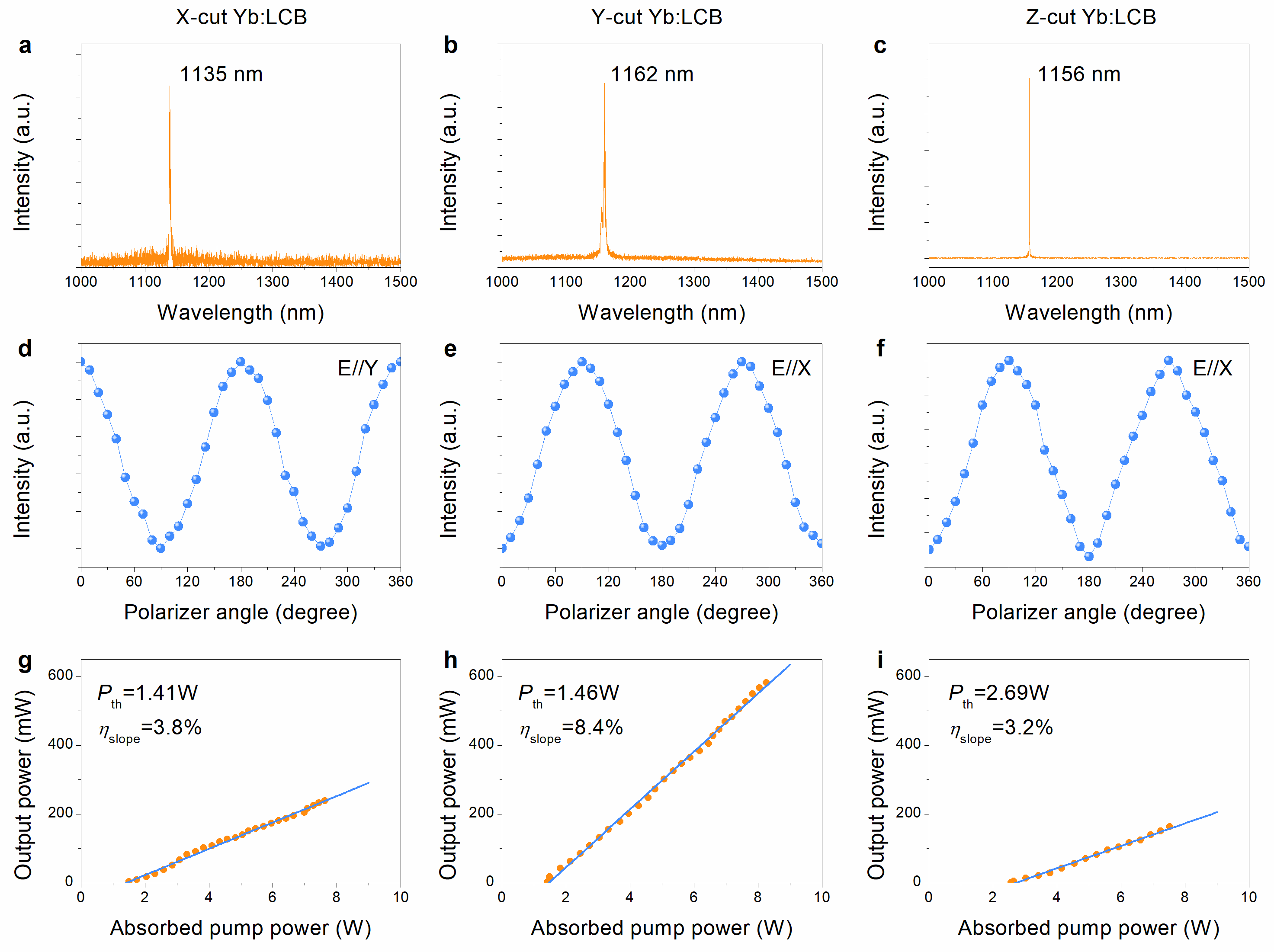


**Supplementary Fig. 13 | Laser performances beyond the fluorescence spectrum in X-cut, Y-cut, and Z-cut Yb:LCB crystal.** (a, b, c) laser wavelength, (d, e, f) laser polarization; X-cut Yb:LCB (E//Y), Y-cut Yb:LCB (E//X), Z-cut Yb:LCB (E//X); (g, h, i) laser output power, X-cut Yb:LCB (P_th_=1.41 W, slope efficiency=3.8%), Y-cut Yb:LCB (P_th_=1.46 W, slope efficiency=8.4%), Z-cut Yb:LCB (P_th_=2.69 W, slope efficiency=3.2%).


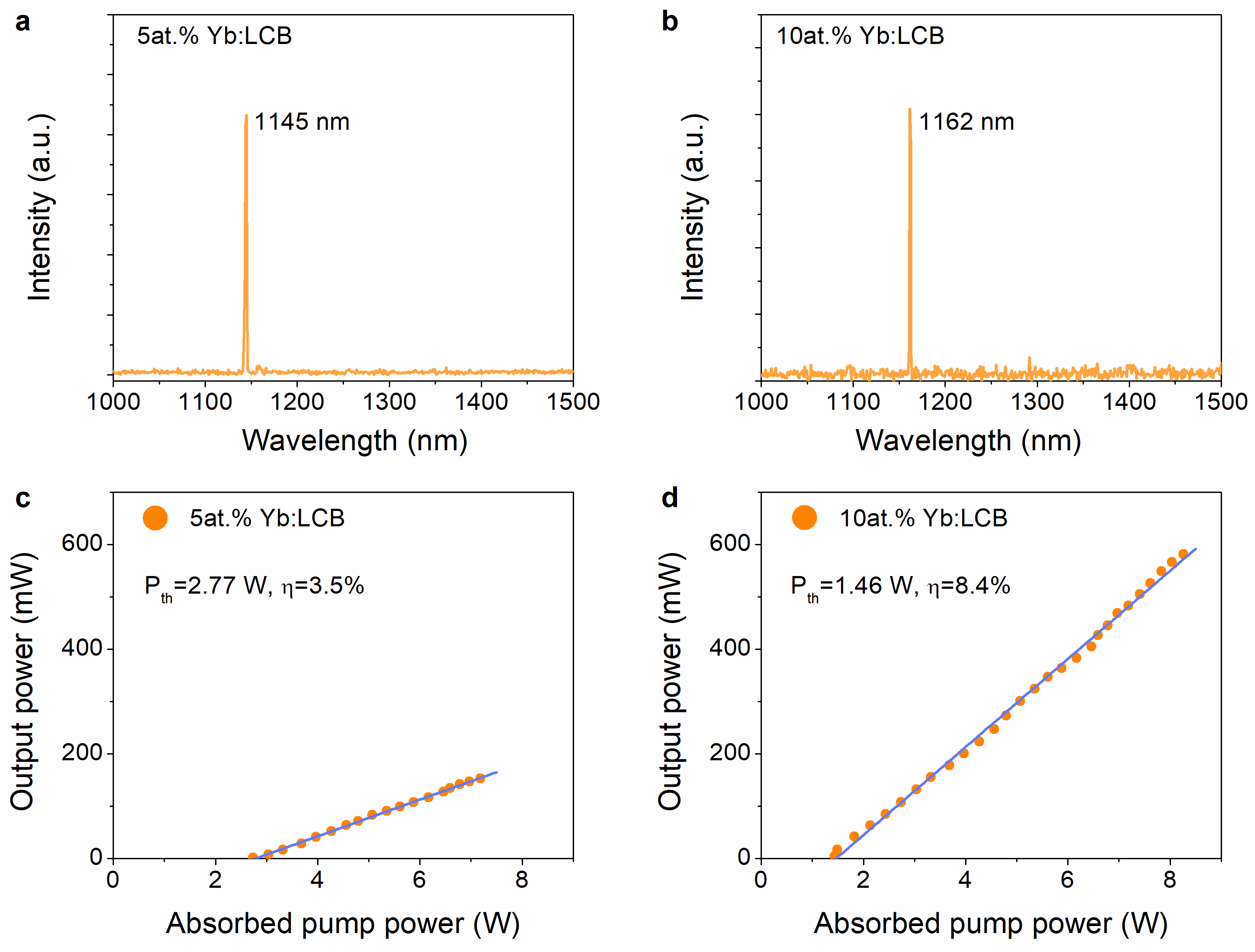


**Supplementary Fig. 14 |** Four-phonon-assisted laser performances of 5at.% Yb:LCB and 10at.% Yb:LCB crystal. (a, b) laser wavelength, (c, d) output power.

We measured the laser performances of 5at.% Yb:LCB and 10at.% Yb:LCB crystal. As shown in **Fig. S14**, 10at.% Yb:LCB is better and exhibits higher laser slope efficiency. We have tried to grow Yb:LCB crystals with high Yb content more than 20at.%. However, it is very difficult to obtain high-quality heavily Yb-doped LCB crystals, because the large ionic radius difference between doped Yb^3+^ and host La^3+^ (Ca^2+^) ions will induce the crystal crack. Therefore, we used 10at.% Yb:LCB crystal in our experiments.


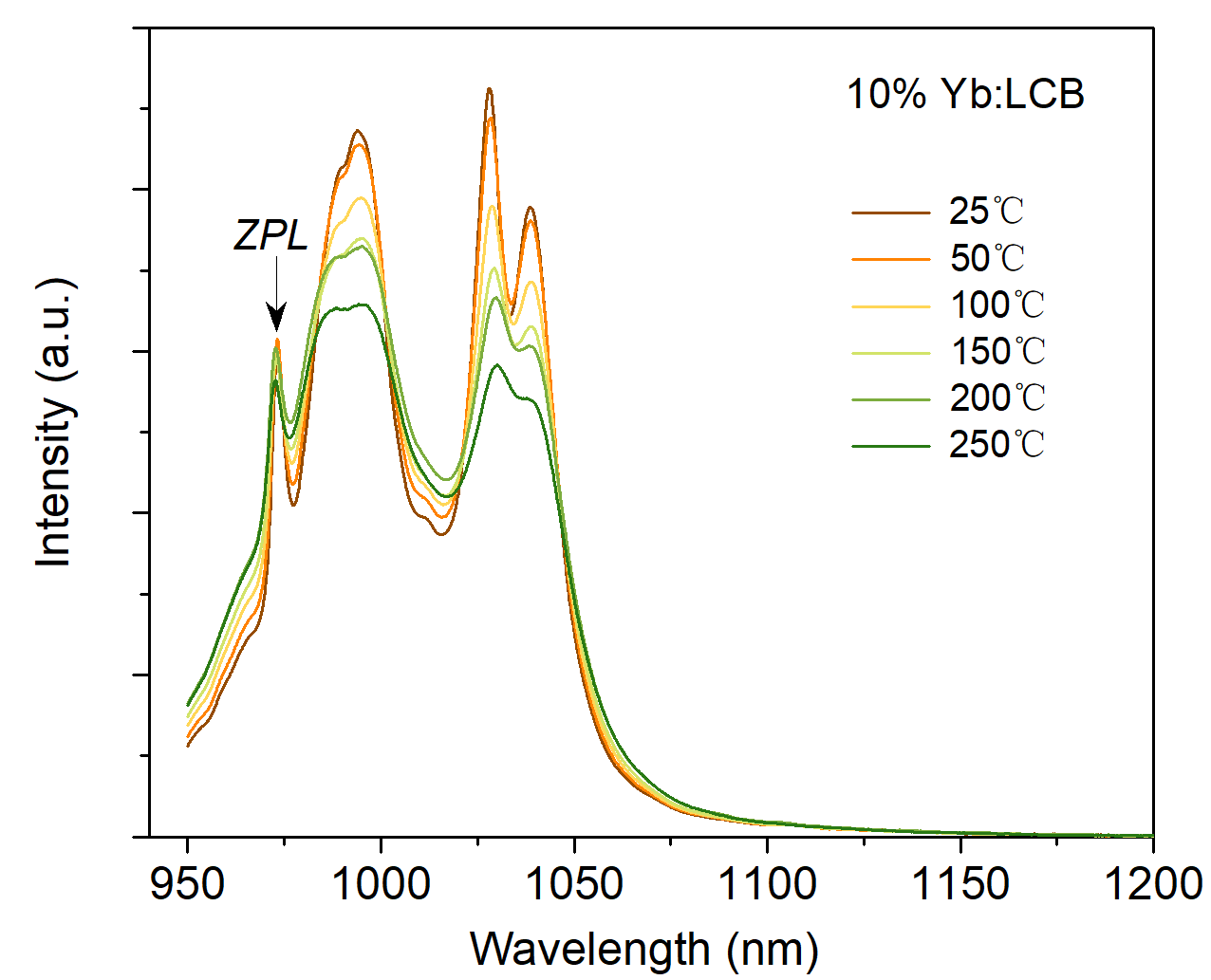


**Supplementary Fig. 15 |** High temperature fluorescence spectrum of 10at. % Yb:LCB crystal. One can see that there is no any fluorescence signal at 1150-1200 nm at high temperatures.


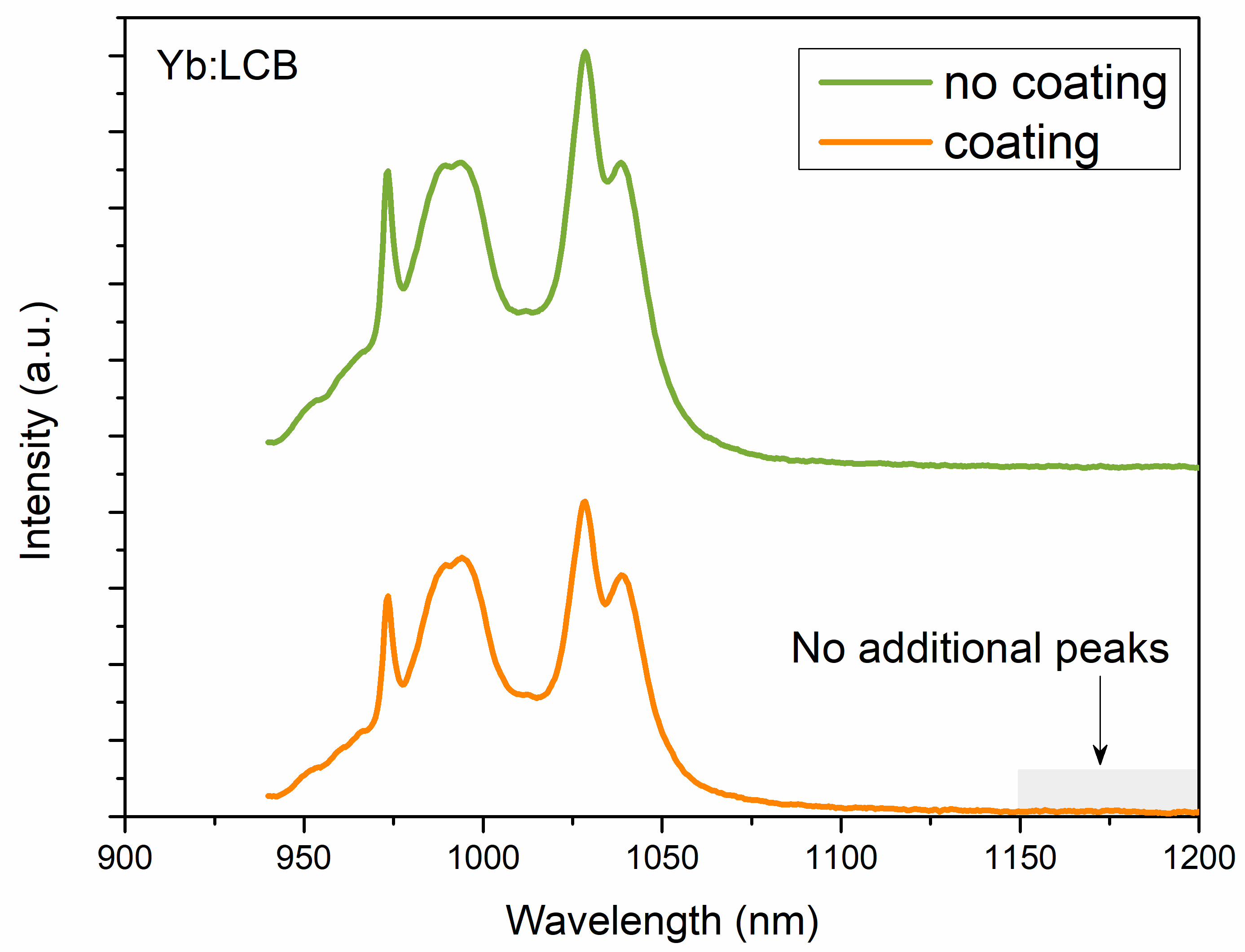


**Supplementary Fig. 16 |** Fluorescence spectrum of coated and uncoated Yb:LCB crystal. One can see that there is no any additional fluorescence signal at 1150-1200 nm with coating.

**
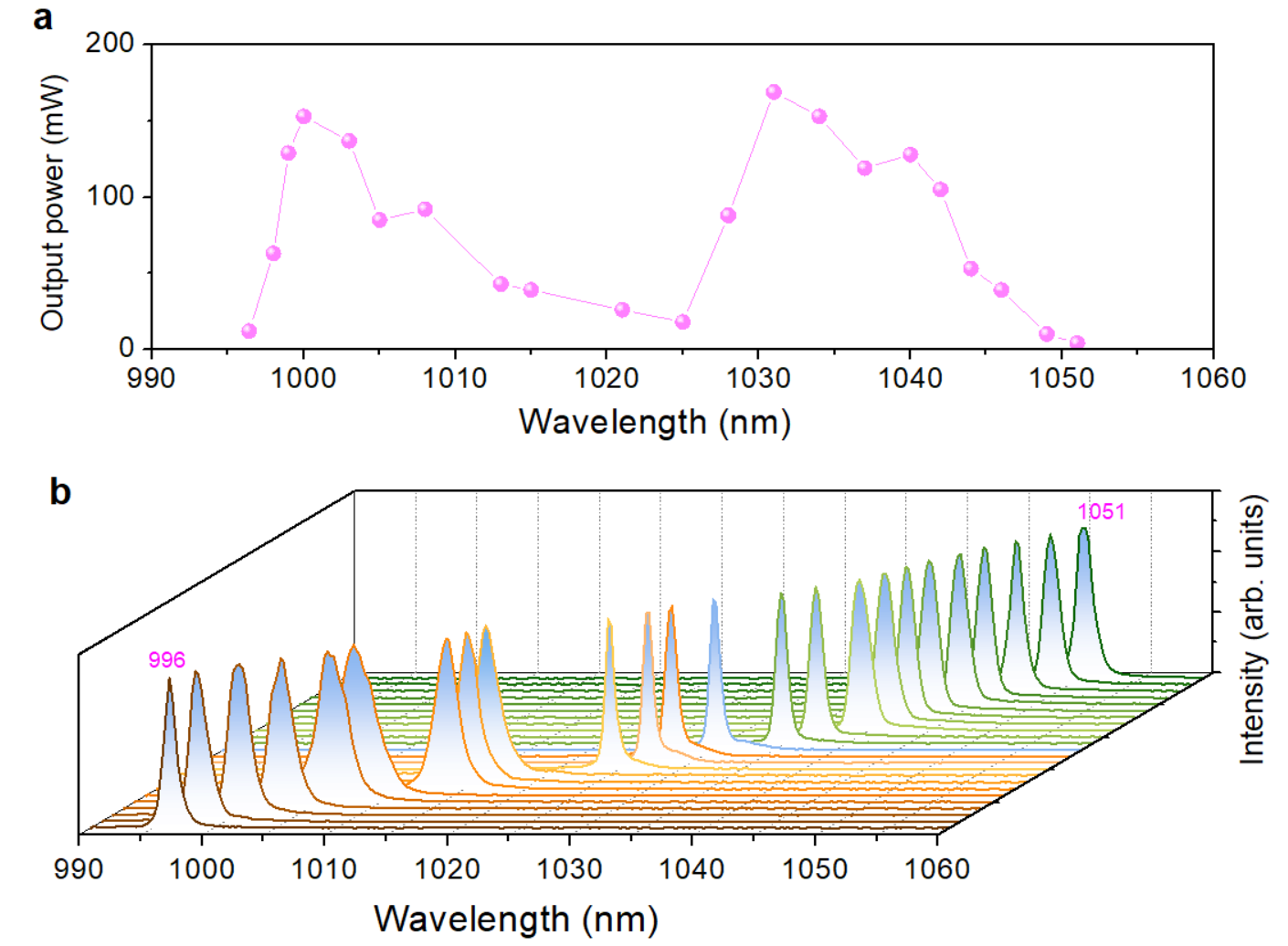
**

**Supplementary Fig. 17 |** Tunable laser output power (a) and laser spectrum (b) at 996-1051 nm in Yb:LCB crystal.

A plano-concave cavity is utilized with input mirror M1 and output coupler M2. The coatings on the mirrors are designed as: M1 is coated with high-transmission (HT, T > 99%) at 976 nm and high-reflection (HR) at 995 - 1100 nm, M2 is partially transmittance at 995 - 1100 nm (T_oc_ = 0.2%). A quartz birefringent filter is inserted along Brewster’s angle. By rotating the BF, a continuously tunable laser from 996 to 1051 nm can be obtained. There are two peaks around 1000 and 1030 nm on the tunable power curve with maximum output power of 153 mW at 1034 nm.

8
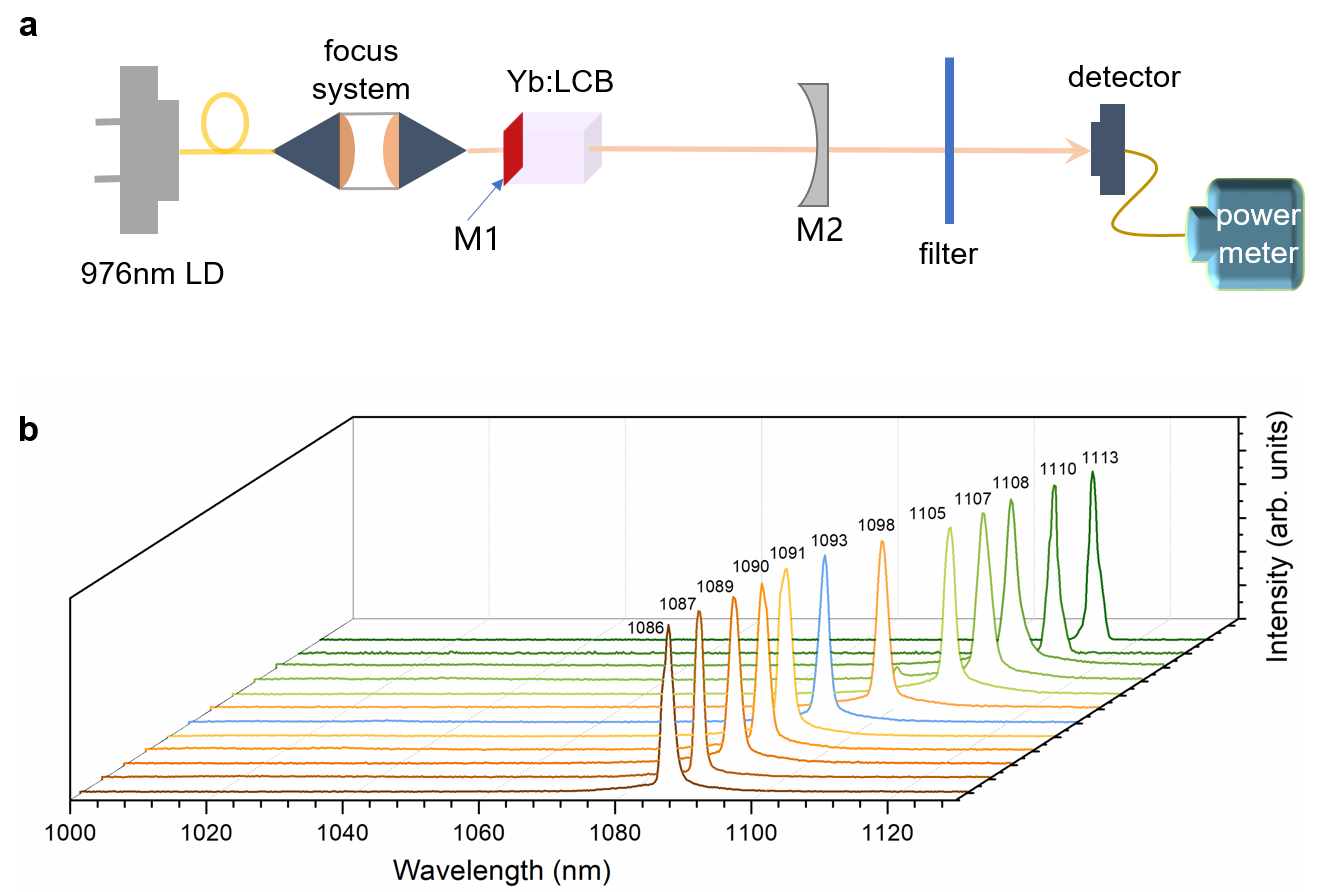


**Supplementary Fig. 18 |** (a) Experimental setup for straight cavity laser beyond the fluorescence spectrum. [M1 coating: 1000-1060 nm HT, 1080-1200 nm HR; M2 mirror: 1000-1060 nm HT, 1080-1200 nm, T_oc_=0.1%] (b) Laser wavelength with the involved phonon number n=3.

We have tried to realize the tunable laser generation in Yb:LCB crystal with high phonon numbers involved. A straight cavity was used. The input mirror was coated on the front face of laser crystal and a HR mirror was used as the output coupler.

In this straight cavity, we realized laser generation from 1086 to 1113 nm by adjusting the incident absorbed pump power from 3 to 8 W (**Fig. R7**). This wavelength range was enveloped by the phonon states of “quasi-free-oxygen” motif coupled to electronic levels with phonon number n=3. The wavelength shift can be attributed to a synergistic effect of temperature-induced phonon frequency change, zero-phonon line shift, and possible spatial hole burning effect. According to these results, the tunable laser wavelengths with higher phonon numbers involved (n≥4) can be expected in Yb:LCB crystal. However, at present, it is still a great challenge owing to its reduced electron-phonon coupling intensity compared to Yb:YCOB crystal, as indicated by a small Huang-Rhys factor.


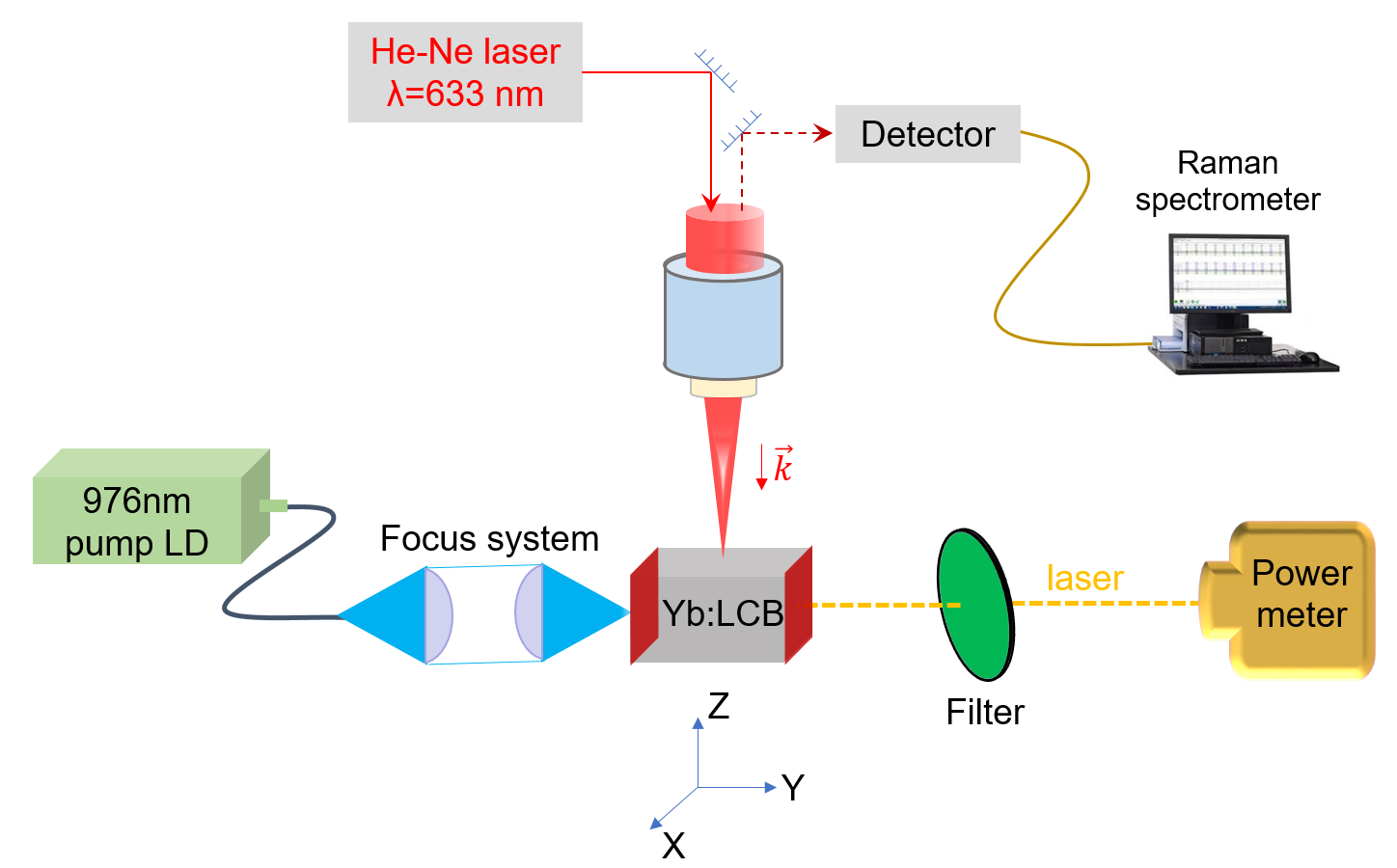


**Supplementary Fig. 19 |** The schematic diagram of in-situ Raman spectrum measurement.


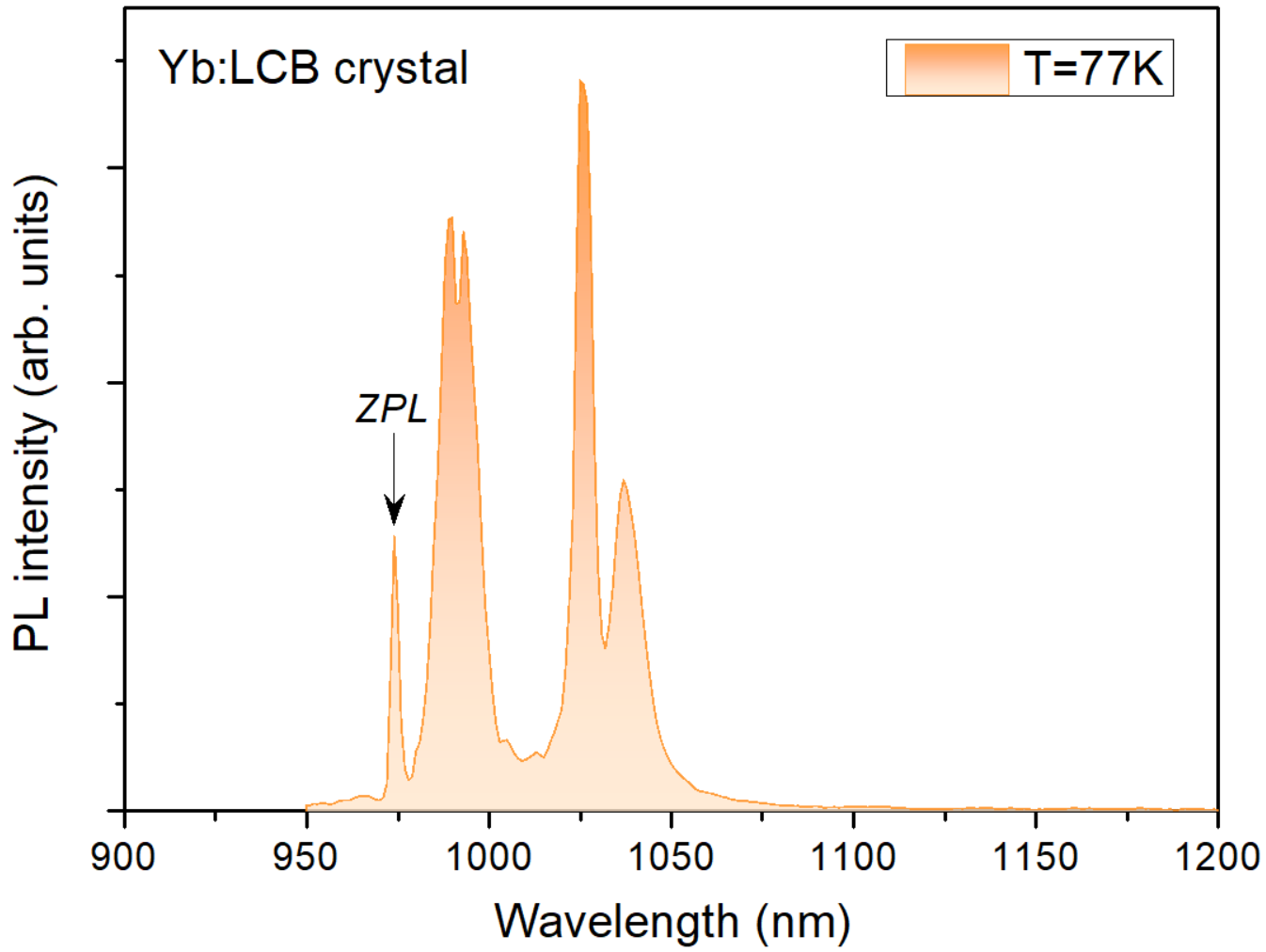


**Supplementary Fig. 20 |** The low-temperature fluorescence of Yb:LCB excited by a 940 nm LD.


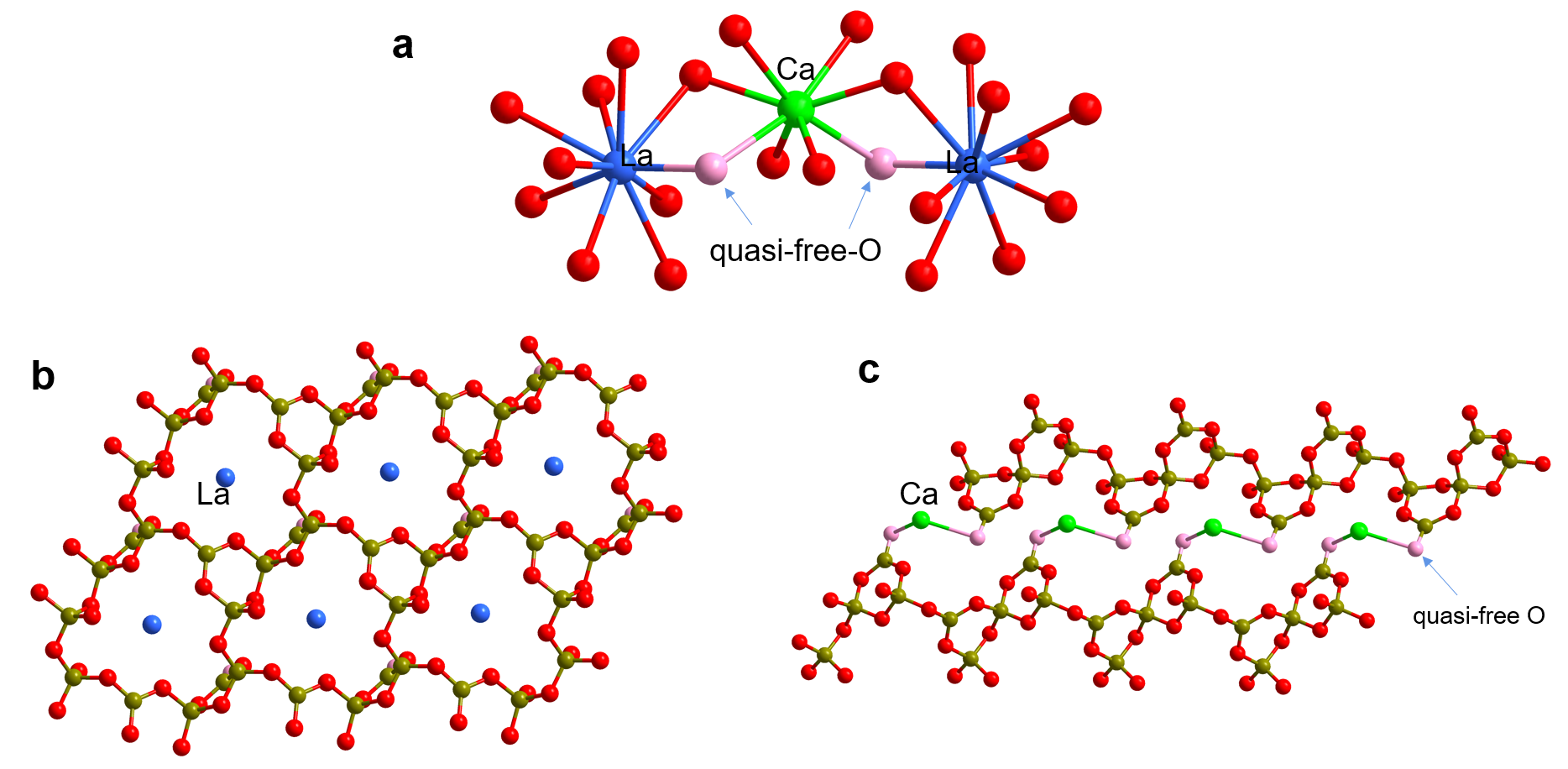


**Supplementary Fig. 21 |** (a) Coordination environment of La^3+^ and Ca^2+^ ion. (b, c) Crystal structure of LCB at La^3+^ site and Ca^2+^ site.

**Supplementary** **Table S1**. A comprehensive analysis for multiphonon-assisted laser wavelength in Yb:LCB crystal.

| Zero-phonon line  (nm) | phonon frequency  (cm^-1^) | phonon number  (n) | frequency shift  (cm^-1^) | multiphonon-assisted  laser wavelength  (nm) |
| --- | --- | --- | --- | --- |
| 976 | 335 | 1 | 335 | 1009 |
| 976 | 424 | 1 | 424 | 1018 |
| 976 | 335 | 2 | 670 | 1044 |
| 976 | 424 | 2 | 848 | 1064 |
| 976 | 335 | 3 | 1005 | 1082 |
| 976 | 424 | 3 | 1272 | 1114 |
| 976 | 335 | 4 | 1340 | 1123 |
| 976 | 424 | 4 | 1696 | 1170 |
| 976 | 335 | 5 | 1675 | 1167 |
| 976 | 424 | 5 | 2120 | 1247 |
| 976 | 335 | 6 | 2010 | 1214 |
| 976 | 424 | 6 | 2544 | 1298 |
| 976 | 335 | 7 | 2345 | 1266 |
| 976 | 424 | 7 | 2968 | 1374 |
| 976 | 335 | 8 | 2680 | 1322 |
| 976 | 424 | 8 | 3392 | 1459 |
| 976 | 335 | 9 | 3015 | 1383 |
| 976 | 424 | 9 | 3816 | 1555 |
| 976 | 335 | 10 | 3350 | 1450 |
| 976 | 424 | 10 | 4240 | 1665 |
